# MnSOD Upregulation Induces Autophagic Programmed Cell Death in Senescent Keratinocytes

**DOI:** 10.1371/journal.pone.0012712

**Published:** 2010-09-14

**Authors:** Emeric Deruy, Karo Gosselin, Chantal Vercamer, Sébastien Martien, Fatima Bouali, Christian Slomianny, Julie Bertout, David Bernard, Albin Pourtier, Corinne Abbadie

**Affiliations:** 1 Université Lille Nord de France, Lille, France; 2 CNRS, UMR8161, Institut de Biologie de Lille, Lille, France; 3 USTL, Villeneuve d'Ascq, France; 4 UDSL, Lille, France; 5 Institut Pasteur de Lille, Lille, France; 6 INSERM, U600, Laboratoire de Physiologie Cellulaire, Villeneuve d'Ascq, France; 7 CNRS, UMR5238, Centre Léon-Bérard, Lyon, France; Roswell Park Cancer Institute, United States of America

## Abstract

Senescence is a state of growth arrest resulting mainly from telomere attrition and oxidative stress. It ultimately leads to cell death. We have previously shown that, in keratinocytes, senescence is induced by NF-kappaB activation, MnSOD upregulation and H_2_O_2_ overproduction. We have also shown that senescent keratinocytes do not die by apoptosis but as a result of high macroautophagic activity that targets the primary vital cell components. Here, we investigated the mechanisms that activate this autophagic cell death program. We show that corpses occurring at the senescence plateau display oxidatively-damaged mitochondria and nucleus that colocalize with autophagic vacuoles. The occurrence of such corpses was decreased by specifically reducing the H_2_O_2_ level with catalase, and, conversely, reproduced by overexpressing MnSOD or applying subtoxic doses of H_2_O_2_. This H_2_O_2_-induced cell death did occur through autophagy since it was accompanied by an accumulation of autophagic vesicles as evidenced by Lysotracker staining, LC3 vesiculation and transmission electron microscopy. Most importantly, it was partly abolished by 3-methyladenine, the specific inhibitor of autophagosome formation, and by anti-Atg5 siRNAs. Taken together these results suggest that autophagic cell death is activated in senescent keratinocytes because of the upregulation of MnSOD and the resulting accumulation of oxidative damages to nucleus and mitochondria.

## Introduction

*In vivo* as *in vitro*, normal human cells have a limited lifespan. After having performed a certain number of divisions, they enter a special state termed senescence [1] which is the consequence of both the telomere erosion occurring at each replication cycle and of oxidative damages which increase with time. Senescent cells are cell-cycle arrested and display numerous morphological, metabolic and genetic changes [2], [3]. Recently, it was shown by us and others that senescence is associated with an increase in macroautophagic activity [4]–[6].

The macroautophagic process, here referred as autophagy, starts by encircling a damaged cell component inside a double membrane. The autophagosome resulting from the closure of this membrane then fuses with endosomes and lysosomes to form an autophagolysosome, inside which the sequestered material is submitted to an acidic pH and to the activity of various hydrolytic enzymes. The recognition of the altered material, and the formation, migration and fusion of the autophagic vacuoles at different stages involve about thirty Atg genes [7]–[9].

We have shown that the autophagic activity associated with senescence of normal human epidermal senescent keratinocytes (NHEKs) occurs at a so high level that it targets the main vital cells components and ultimately leads to cell death [4]. The dying senescent keratinocytes are characterized by an accumulation of a huge quantity of autophagic vacuoles and a particular intracellular organisation. Their cytokeratin network develops to form a cage that partitions the intracellular space in two areas: a cortical one completely devoid of organelles and a central one in which are concentrated mainly all the organelles including the nucleus and all the autophagic vacuoles. The nuclei and mitochondria localized in this central area display various degrees of morphological damages, suggesting they are degraded therein [4].

Since escaping senescent-cell death could be a requisite step in neoplastic transformation, it is important to establish which the inducers of the autophagic programmed cell death encountered by senescent cells are. The purpose of this study was hence to analyse the type of damages undergone by senescent cells and to investigate whether these damages are responsible for the activation of the autophagic process at a lethal level. We had established in a previous work that keratinocyte senescence partly results from an accumulation of hydrogen peroxide (H_2_O_2_) due to the up-regulation of MnSOD by NF-kappaB transcription factors. MnSOD is a mitochondrial redox enzyme that dismutates O_2_^.-^ in H_2_O_2_; hence, since H_2_O_2_-degrading enzymes such as catalase or glutathione peroxidase are not upregulated during keratinocyte senescence in coordination with MnSOD, the increased MnSOD expression leads to H_2_O_2_ accumulation [10]. Here, we hypothesized and demonstrate that, in addition to be involved in the establishment of the senescent phenotype itself, this H_2_O_2_ accumulation sufficiently damages nuclei and mitochondria to target them for massive autophagic elimination, hence leading to the death of senescent cells.

## Methods

### Cell culture

Normal human epidermal keratinocytes (NHEK) were purchased from Clonetics (CC-2501). We used cells from 6 different female donors of different race and age. Cells were obtained anonymously and informed consent of each skin donor was obtained by the supplier. Cells were grown at 37°C in an atmosphere of 5% CO_2_ in the KGM-2 BulletKit medium consisting of modified MCBD 153, with 0.15 mM calcium, supplemented with bovine pituitary extract, EGF, insulin, hydrocortisone, transferrin and epinephrin (CC-3107, Clonetics). Such a serum-free low-calcium medium was shown to minimize keratinocyte terminal differentiation [11]. In all experiments, cells were seeded as recommended by the supplier at 3500 cells/cm^2^. When necessary, they were split at 70% confluence. The number of population doublings (PD) was calculated at each passage by means of the following equation: PD = ln(number of collected cells/number of plated cells)/ln2.

### Western-blotting

Cells were lysed in the following solution: Hepes 27.5 mM pH 7.6, urea 1.1 M, NaCl 0.33 M, EGTA 0.1 M, EDTA 2 mM, KCl 60 mM, DTT 1 mM and NP40 1.1%. The total protein concentration was measured with the Bio-Rad protein assay. Proteins were resolved by SDS-PAGE and transferred to nitrocellulose membranes (Hybond-C extra, Amersham). Equal loading was verified after a Ponceau Red coloration of the membranes. Primary antibody used was an anti human MnSOD sheep IgG (Calbiochem), anti human Atg5 rabbit antibody (Cell Signalling) or anti human GAPDH mouse monoclonal antibody (Chemicon International). Secondary antibodies used were peroxidase-conjugated (Jackson ImmunoResearch Laboratories). Peroxidase activity was revealed using a ECL (enhanced chemiluminescence) or ECL advance kit (Amersham Biosciences).

### Immunofluorescence

For detection of MnSOD, AIP bridges and LAMP1, cells were fixed with 4% paraformaldehyde in PBS, permeabilized with 0.2% Triton-X100. For 8oxoG immunodetection, cells were fixed in 4% paraformaldehyde for 15 mn at 4°C, dehydrated in 70% and 95% methanol for 3 mn followed by 99% methanol for 30 mn at −20°C. Finally cells were rehydrated by 3 mn incubation at −20°C in 95% and 70% methanol, and washed 3 times in PBS. Slides were incubated with a primary antibody: anti-MnSOD (Chemicon), anti-8oxoG (Trevigen), anti-LAMP1 (Santa Cruz) and anti-AIP that are mouse monoclonal antibodies produced by [12]. Cells were then washed 3 times with PBS and incubated with the secondary antibody: Rhodamine Red-conjugated anti-Mouse IgG or Rhodamine Red-conjugated anti-Rabbit IgG (Jackson ImmunoResearch Laboratories). Nuclei were stained by Hoechst 33258 at 1 µg/ml for 3 mn.

### Fluorescent co-staining of mitochondria and lysosomes

Lysotracker green and Mitotracker red were from Molecular Probes. Living cells were incubated with Lysotracker green directly added in the cell culture medium at 37°C at 100 nM for 2 hrs, or with Mitotracker red at 25 nM for 30 mn. Nuclei were stained with the vital Hoechst 33342 at 1 µg/ml for 10 mn at 37°C.

### Transmission electron microscopy

Cell pellets were fixed with 2.5% glutaraldehyde in 0.1 M cacodylate buffer, pH 7.4 for at least 30 min at 4°C. After fixation, the specimens were thoroughly washed in 0.1 M cacodylate buffer and then postfixed with 1% osmium tetroxide in the same buffer for 1 h at room temperature, stained *en bloc* with 2% uranyl acetate in distilled water for 15 min, dehydrated in graded acetonitrile, and embedded in Epon. Ultrathin sections (80–100 nm thick) mounted on 150-mesh grids were stained with 2% uranyl acetate solution and Reynolds lead citrate solution [13]. The electron micrographs were taken with a Hitachi H600 electron microscope at 75 kV.

### SA-beta-Galactosidase assays

SA-beta-Gal assays were performed as described by Dimri [14].

### Annexin-V assays

Cells were processed with an Annexin-V-Alexa 568 kit (Roche, Calbiochem) according to manufacturer's recommendations.

### Flow cytometry measurement of ROS levels

ROS levels were measured using non-fluorescent H_2_-DCFDA (2′,7′-dichlorofluorescein diacetate) (D399, Molecular Probes) which diffuses across membranes and is oxidized to fluorescent DCF. Cells were rinsed in PBS, incubated with H_2_-DCFDA diluted in medium at 5 µM for 30 min at 37°C. After that, cells were washed, trypsinized, and re-suspended in pre-warmed PBS at 37°C. They were analyzed for forward and side scatter factor values and fluorescence intensity using a flow cytometer (Coulter EPICS XL-MCL) with FITC filters. The results were analyzed with the WinMDI 2.9 software.

### Flow cytometry measurement of acidic vesicles

Acidic vesicles levels were measured using Lysotracker green (Molecular Probes). Cells were incubated with Lysotracker directly added in the cell culture medium at 37°C at 200 nM for 15 min. After that, cells were washed, trypsinized, and re-suspended in pre-warmed PBS at 37°C. They were analyzed for forward and side scatter factor values and fluorescence intensity using a flow cytometer (Coulter EPICS XL-MCL) with FITC filters. The results were analyzed with the WinMDI 2.9 software.

### Flow cytometry sorting of senescent cells. Antioxidant treatment

NHEKs were analyzed on a BD FACS Aria and the subpopulation with the ad hoc forward and side scatter factor values was electrostatically sorted in air, collected in complete culture medium and put again in culture. After plating, cells were treated either by Catalase (Sigma, C1345) or PEG-catalase (Sigma, C4963) diluted in PBS and directly added in the culture medium at different final concentrations.

### Adenoviral vector encoding MnSOD

The human MnSOD cDNA was obtained after retrotranscription, amplified by PCR and inserted into the pcDNA3.1 as previously described [15]. The MnSOD cDNA was then digested by EcoRI and inserted into the pAdCMV2 vector between the XbaI sites after filling with Klenow polymerase. Recombinant adenovirus vectors (AdMnSOD) were obtained by homologous recombination in *E. coli* BJ5183 as described in [16] (details are available on request). Viral stocks were amplified after infection of N52.E6 cells [17]. Recombinant adenoviruses were purified using ViraBind Adenovirus purification kit (Cell Biolabs Inc., San Diego, CA) and titrated using Adeno-X rapid titer kit (BD Biosciences Clontech, Palo Alto, CA, USA). Cells were infected by adding virus stocks directly to the culture medium at an input multiplicity of 200 viral particles/cell.

### Inhibition of autophagy by RNA interference

NHEK at exponential growth phase were plated at 70,000 cells per well in six-well plates. The day of transfection, culture media were renewed and siRNA mixtures prepared using PrimeFect siRNA Transfection Reagent diluted 1/100 in PrimeFect diluent (purchased from Lonza) and incubated 15 minutes at room temperature before adding to cells. Inhibition of Atg5 expression was performed using 25 or 50 nM of a pool of 4 siRNA (siGENOME SMARTpool, Dharmacon - GGAAUAUCCUGCAGAAGAA - CAUCUGAGCUACCCGGAUA - GACAAGAAGACAUUAGUGA - CAAUUGGUUUGCUAUUUGA). Control transfection was performed using a non targeting siRNA pool (siCONTROL Non Targeting siRNA Pool # 2, Dharmacon). Transfections were stopped after 24 hrs by adding fresh culture medium.

### Cell transfection with the mRFP-GFP-LC3 vector

NHEK at exponential growth phase were treated twice by 50 µM H_2_O_2_ at 48 hrs interval and then electroporated with the mRFP-GFP-LC3 [18] or the mRFP-GFP control vector using the Neon transfection system (Invitrogen) according to supplier recommendations. Briefly, 80,000 cells were suspended in 10 µL of R buffer containing 1 µg of plasmid and the electroporation was performed by 2 pulses at 1400 V for 20 ms. After electroporation, cells were plated in complete culture medium on microscopic slides. Forty eight hrs later, they were stained by Hoechst 33342 at 1 µg/ml for 10 mn at 37°C, mounted in PBS without any fixation, and analyzed under a confocal microscope (LSM710, Zeiss).

### Ethics statement

Human cells used in this study provide from people whose informed consent was obtained by the cell supplier (Clonetics) Cells were obtained anonymously. No ethics approval was necessary for the experiments performed therein.

## Results

### Senescent keratinocytes and corpses at the senescent plateau display altered mitochondria and nuclei that colocalize with autophagic vacuoles

To study the inducers of senescent-cell death by macroautophagy, we used normal human epidermal keratinocytes (NHEKs). As we have shown in previous studies, NHEKs cultured in vitro reach a senescence growth plateau after 15–25 population doublings (PDs). At this plateau most of the cells display all the characteristics of senescence: senescence-associated beta-galactosidase activity at pH 6, upregulation of the CKI p16^INK4^, a 5- to 100-fold larger size than young cells, numerous dense particles (probably protein aggregates), several vacuole-like structures of different sizes, and about 20% of polynucleation [4], [10]. A flow cytometry analysis of AnnexinV- and Propidium Iodide-staining revealed that about 20% of cells at the senescence plateau are dying cells or corpses [4]. Microscopically, they are characterized by their round shape and the presence of a central area that was demonstrated by transmission electron microscopy to be delineated by a keratin cage and to be full of autophagic vacuoles [4].

**Figure 1:**
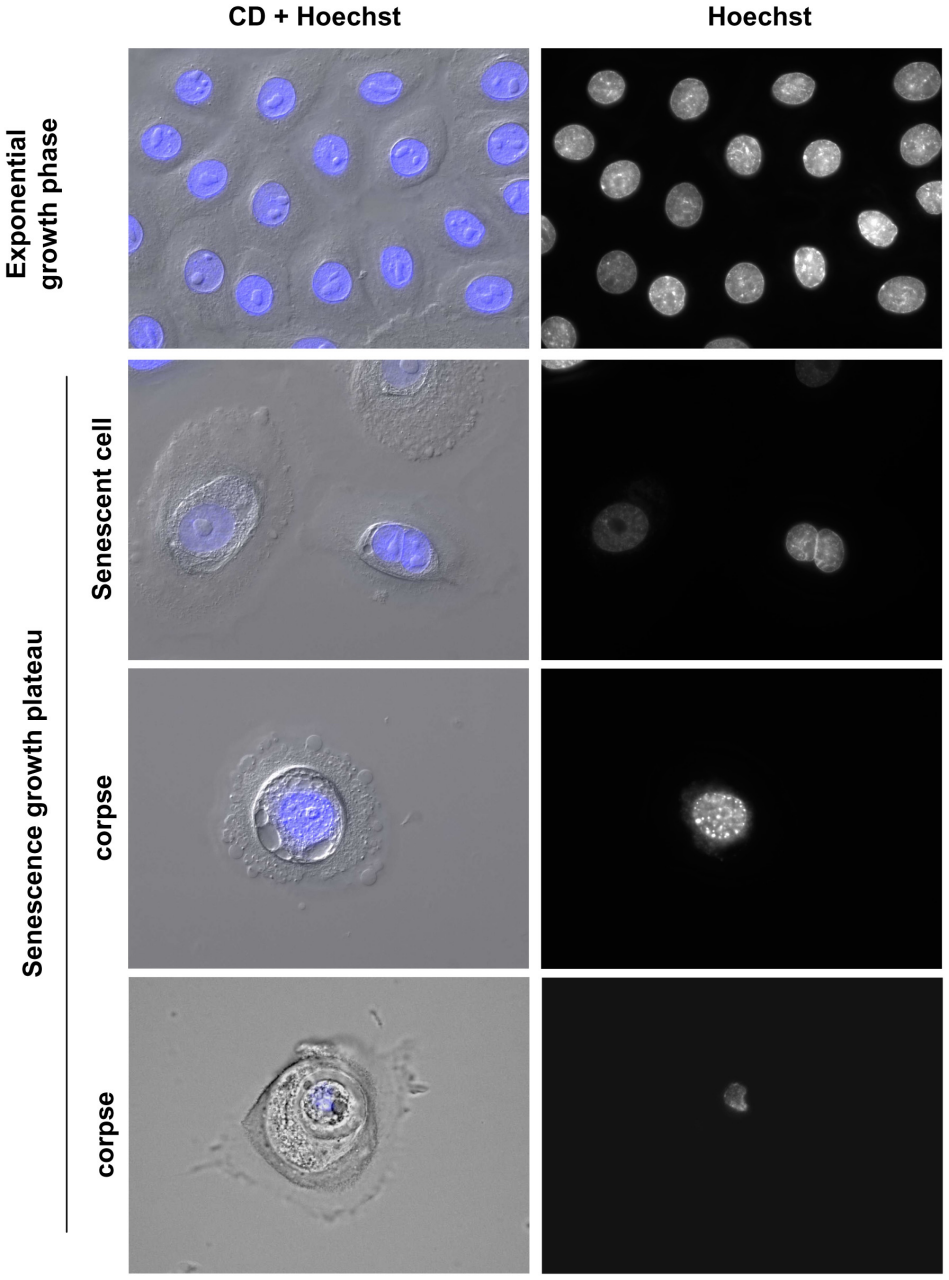
Nuclear damages in senescent keratinocytes and corpses. NHEKs at the exponential growth phase or at the senescence growth plateau were fixed, stained with Hoechst and observed under circular dichroism plus epifluorescent microscopy. Senescent keratinocytes display altered chromatin and are often polynucleated. Corpses are characterized by the presence of a central area always containing a much damaged nucleus. Images are representative of all the senescent cells and corpses visible at the senescence plateau of different cell donors. Scale bar = 20 µM.

In order to make a first investigation of the nuclear damages that could contribute to the induction of autophagic programmed cell death in senescent NHEKs, we performed a Hoechst staining at the senescence plateau. Nuclei of senescent cells are often larger than that of young cells and their chromatin organisation can appear slightly altered (Figure 1). In corpses, the nuclei found in the central area always display a damaged chromatin and are often deformed (Figure 1).

**Figure 2:**
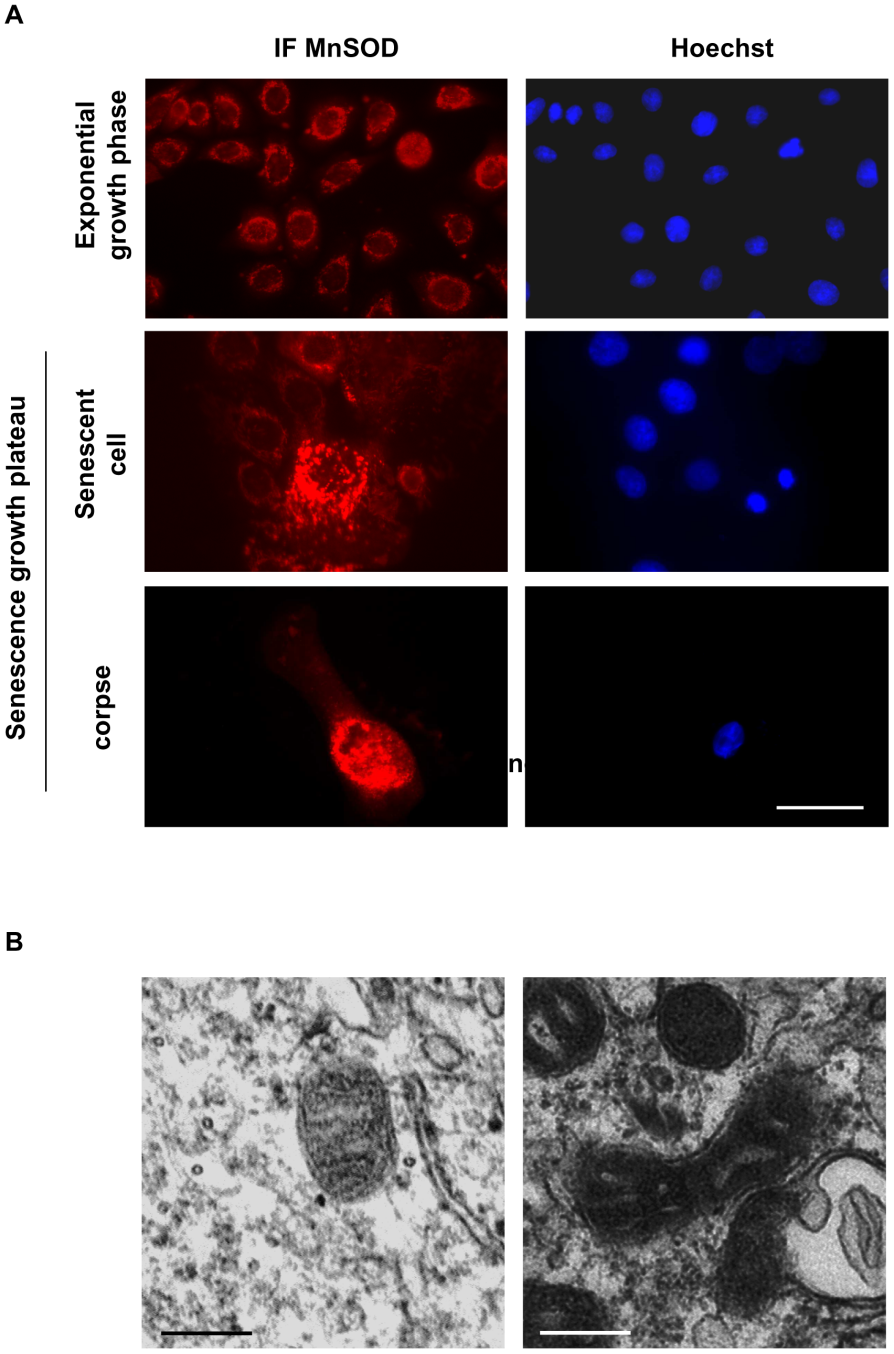
Mitochondrial damages in senescent keratinocytes and corpses. (A) NHEKs at the exponential growth phase or at the senescence plateau were immunostained with anti MnSOD antibodies. In almost all senescent cells, the stained structures (the mitochondria) are vesiculated and agglutinated in the vicinity of the nucleus. In corpses, the stained structures are concentrated in the central area which also contains the altered nucleus. Scale bars = 40 µM. (B) NHEKs at the exponential growth phase or at the senescence plateau were trypsinized and prepared for transmission electron microscopy. Details of mitochondrial morphology are shown. In young cells at the exponential growth phase, mitochondria have a normal morphology, whereas those of senescent cells display very dark and thickened cristae. Scale bars = 0.25 µM.

We also investigated damages to mitochondria in senescent cells and corpses by performing an immunofluorescence against MnSOD, a mitochondrial matrix enzyme. In cells at the exponential growth phase, MnSOD antibodies stained discreet small sticks or vesicles, the typical appearance of mitochondria. With increasing population doublings, the stained structures tend to increase in number, in size, to vesiculate and to delocalize toward the nucleus. In corpses, almost all the stained structures were concentrated inside the central area (Figure 2A). A transmission electron microscopy analysis confirmed the alteration of mitochondria in senescent cells which displayed very dense and thickened cristae (Figure 2B).

**Figure 3:**
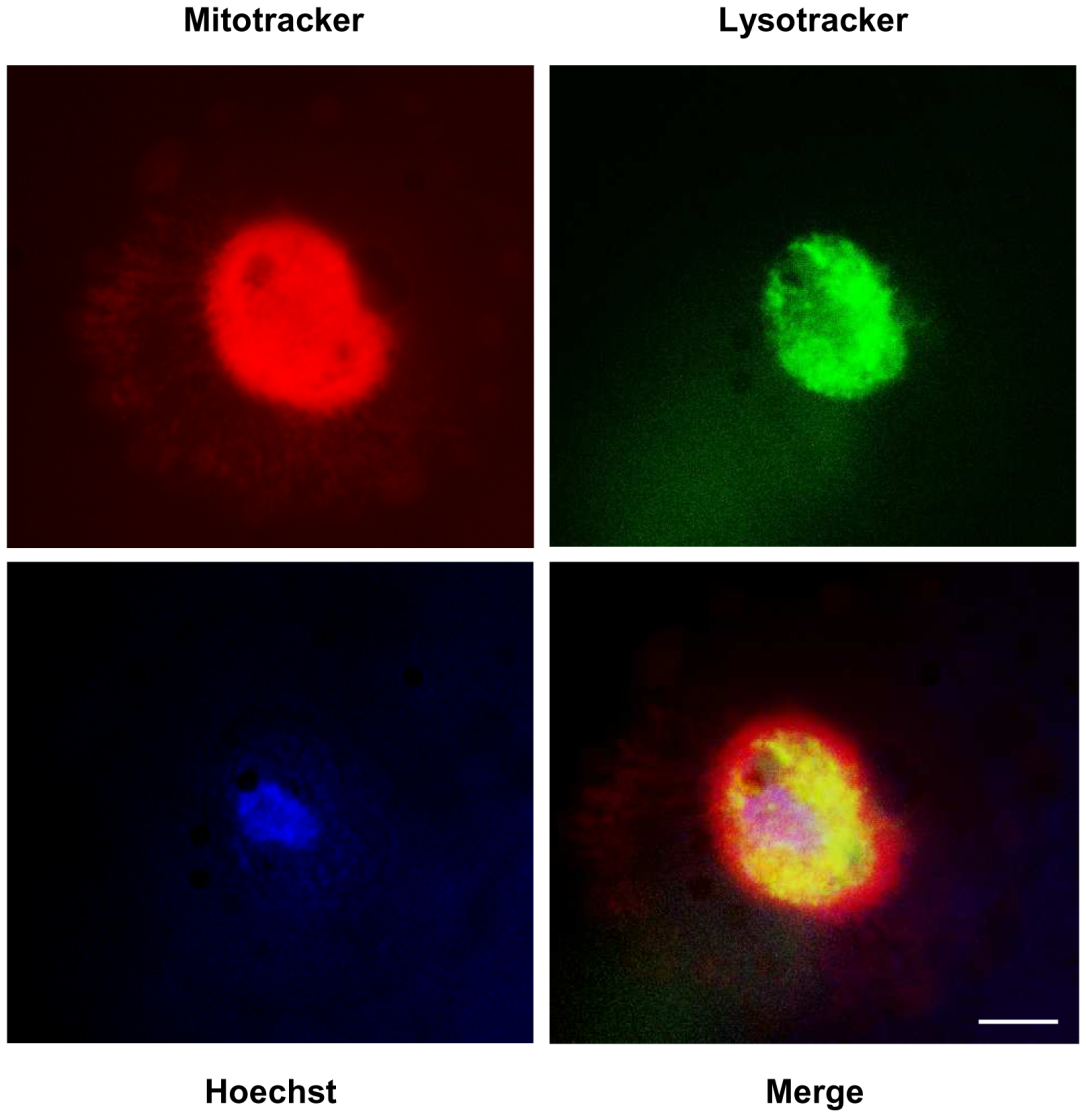
Corpses contain a central area in which mitochondria, nucleus and acidic vacuoles are colocalized. NHEKs at the senescence plateau were triply stained with Mitotracker (red), to stain mitochondria, Lysotracker (green), to stain the acidic organelles (including autophagic vacuoles), and Hoechst (blue) to stain nuclei. A representative image of the staining of corpses is given at high magnification (scale bar = 20 µm). The three stainings co-localize within a central area. The rest of the cell, visible as background in the Mitotracker image, is devoided of any mitochondria or acidic organelles. Note that the Lysotracker staining is vesiculated, as expected, and that the nucleus of the corpse is much damaged.

To confirm the final colocalisation of damaged nuclei and mitochondria with autophagic vacuoles inside the central area of corpses, we performed a triple staining with Hoechst, Mitotracker (a permeant probe that fluoresces in mitochondria upon oxidation) and Lysotracker (a permeant probe that fluoresces in the acidic organelles). The analysis revealed that the three stainings indeed colocalize inside a central area (Figure 3), suggesting that the damaged nucleus and mitochondria are addressed to the central area and are degraded therein by macroautophagy.

### Mitochondria and nuclei of corpses display oxidative damages

**Figure 4:**
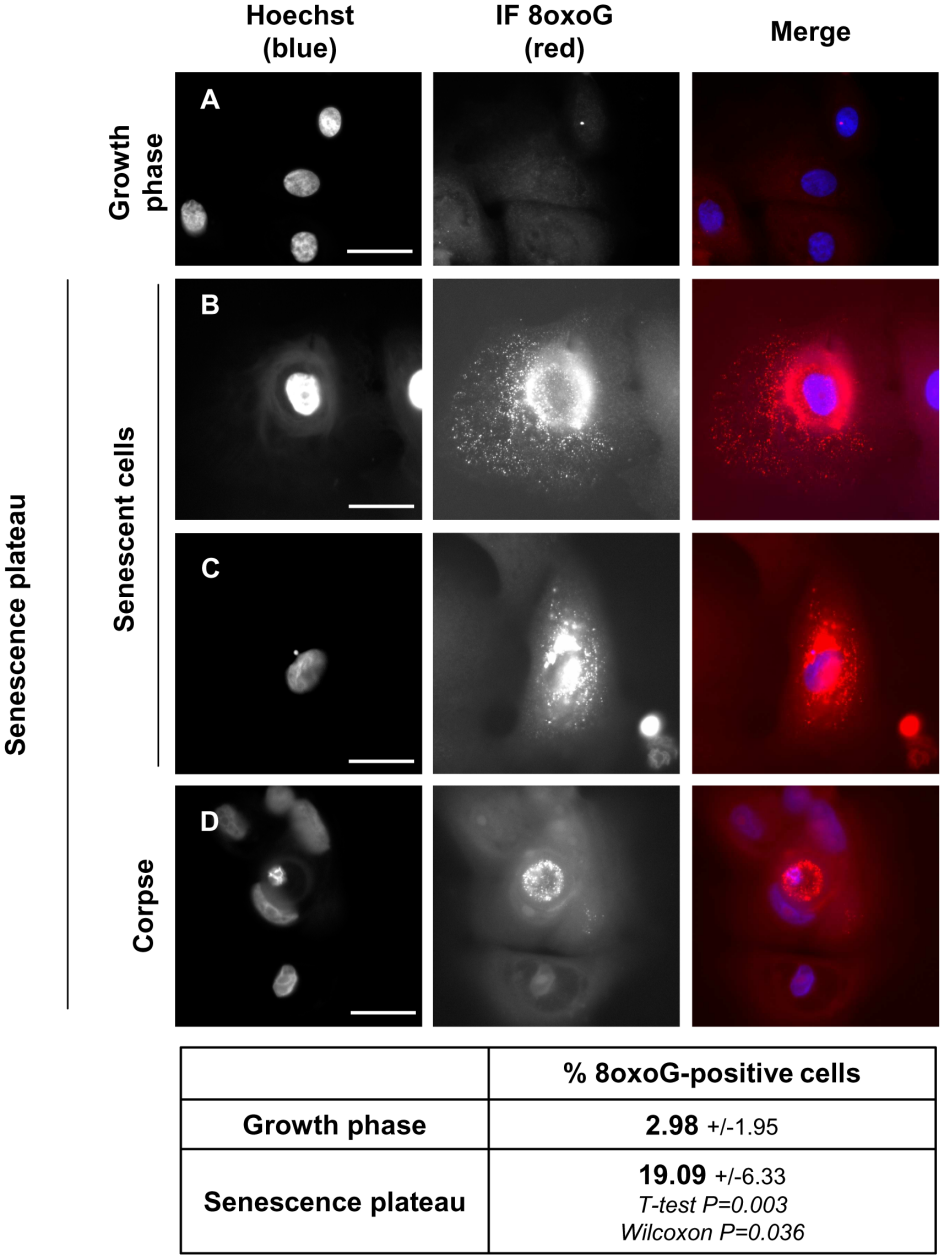
Mitochondria and nuclei of senescent keratinocytes and corpses contain oxidized guanines. NHEKs at the exponential growth phase or at the senescence plateau were processed for immunodetection of 8-oxo-guanines (8oxoG). Different representative images are given. Young cells at the growth phase are negative for 8oxoG (A). Cells at the senescence plateau display different staining patterns, according to their degree of damaging. In senescent cells not much damaged, 8oxoG are mainly localized on punctuated cytoplasmic structures, probably mitochondria (B). When the nucleus is damaged (as indicated by the presence of a micronucleus), 8oxoG are localized inside the nucleus and on punctuated cytoplasmic structures aggregated in the vicinity of the nucleus (C). When the cell is obviously dead and display a very much damaged nucleus inside the central area and another one pushed away by it, 8oxoG are localized inside the central area, inside the nucleus itself as well as inside other punctuated structures (D). Scale bars = 30 µm. The number of 8oxoG-positive cells (including corpses) was manually counted in 5 random microscopic fields amongst a total of 617 young or 123 senescent cells. Values are the mean percentages of 8-oxoG-positive cells +/− SD. Since the data are not strictly normally distributed, P values were calculated using both Student and Wilcoxon tests.

Since oxidative stress is recognized as a main cause of senescence, we postulated that it could lead to the mitochondrial and nuclear damages we evidenced and, hence, to the induction of their degradation by macroautophagy. We therefore searched for the presence of oxidative damages in mitochondria and nuclei of corpses at the senescence plateau. We focussed first on 8-oxo-guanines (8oxoG), the main form of oxidized base that can be detected in nuclear or mitochondrial DNA, as well as in the free nucleotides pool [19]. The quantity of 8oxoG-immunopositive cells dramatically increased from about 3% in cultures at the exponential growth phase to about 20% at the senescence plateau (Figure 4). In senescent cells, the fluorescence was mainly localized on cytoplasmic punctuated structures clustered around the nucleus, probably the damaged mitochondria (Figure 4B and C). In corpses, some staining was observed in the central area both on the nucleus when it appeared damaged, as well as on smaller components, probably damaged mitochondria (Figure 4D).

**Figure 5:**
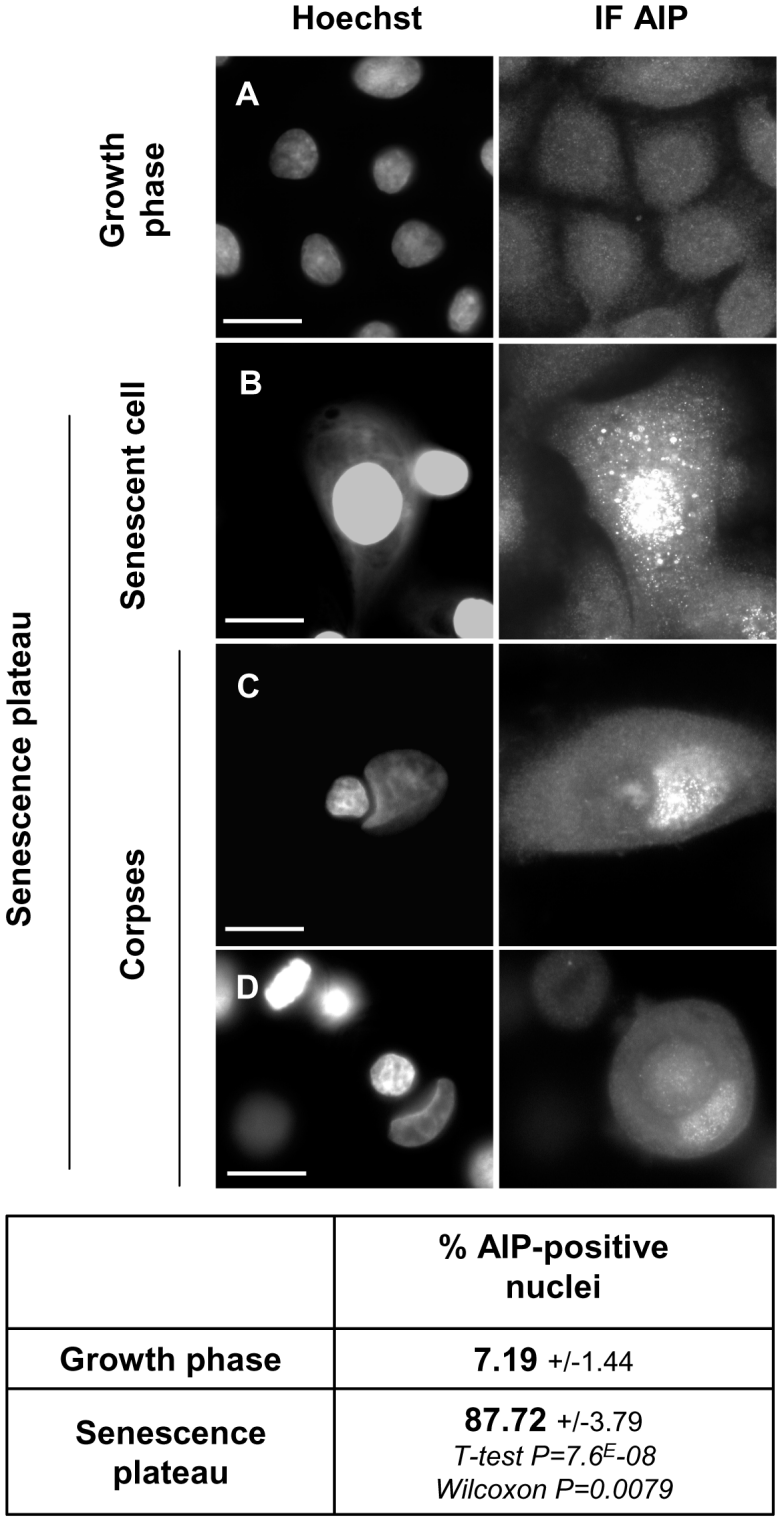
Mitochondria and nuclei of senescent keratinocytes and corpses contain oxidized lipids and proteins. NHEKs at the exponential growth phase or at the senescence plateau were processed for immunodetection of amino-imino-propene bridges (AIP). Young cells at the growth phase are negative for AIP (A). In senescent cells and corpses, AIP are found mainly in the nucleus and in some punctuated cytoplasmic structures (B and C). When the nucleus is localized in the autophagic central area, the staining disappears (C and D). Scale bars = 30 µm. The number of cells (including corpses) with AIP-positive nuclei was manually counted in 5 random microscopic fields amongst a total of 2068 young and 1040 senescent cells. Values are the mean percentages of cells with AIP-positive nuclei +/− SD. Since the data are not strictly normally distributed, P values were calculated using both Student and Wilcoxon tests.

We also examined 1-amino-3-iminopropene (AIP) bridges, an oxidative damage resulting from the reaction of primary amino groups of proteins with malondialdehyde, an end product of lipid peroxidation [12]. Almost all cells in senescent cultures were AIP-positive (Figure 5). The staining was mainly localized in nuclei and also on punctuated cytoplasmic structures, probably mitochondria (Figure 5B and C). In corpses, the nuclei kept outside the central area were AIP-positive, whereas those inside the central area were negative (Figure 5C and D), suggesting that AIP bridges are enzymatically degraded in the central area.

Taken together, these results indicate that mitochondria and nuclei of senescent cells are indeed affected by oxidative damages. They are found in this state inside the central area of corpses in close contact with autophagic vacuoles, suggesting that autophagic programmed cell death of senescent cells could be initiated as a result of their oxidative damage.

### Autophagic programmed cell death is initiated in senescent keratinocytes by MnSOD overexpression and H_2_O_2_ accumulation

To further demonstrate that autophagic cell death is induced in senescent cells following their oxidative damage, we provoked an oxidative stress in young cells that mimics that occurring during senescence and examined whether it induces autophagic cell death. We had previously established that the oxidative stress associated with NHEK senescence results from an activation of NF-kappaB transcription factors, that upregulate the expression of the manganese superoxide dismutase (MnSOD), a mitochondrial enzyme of the redox control, whose increased activity leads to increased hydrogen peroxide (H_2_O_2_) production [10]. Figure 2A confirms the accumulation of MnSOD in senescent cells versus young ones, and indicates in addition that MnSOD is also highly expressed in corpses. The accumulation of ROS in senescent cells was confirmed by a flow cytometry analysis using H_2_-DCFDA, a cell permeant fluorigenic probe that fluoresces upon oxidation (Figure S1).

**Figure 6:**
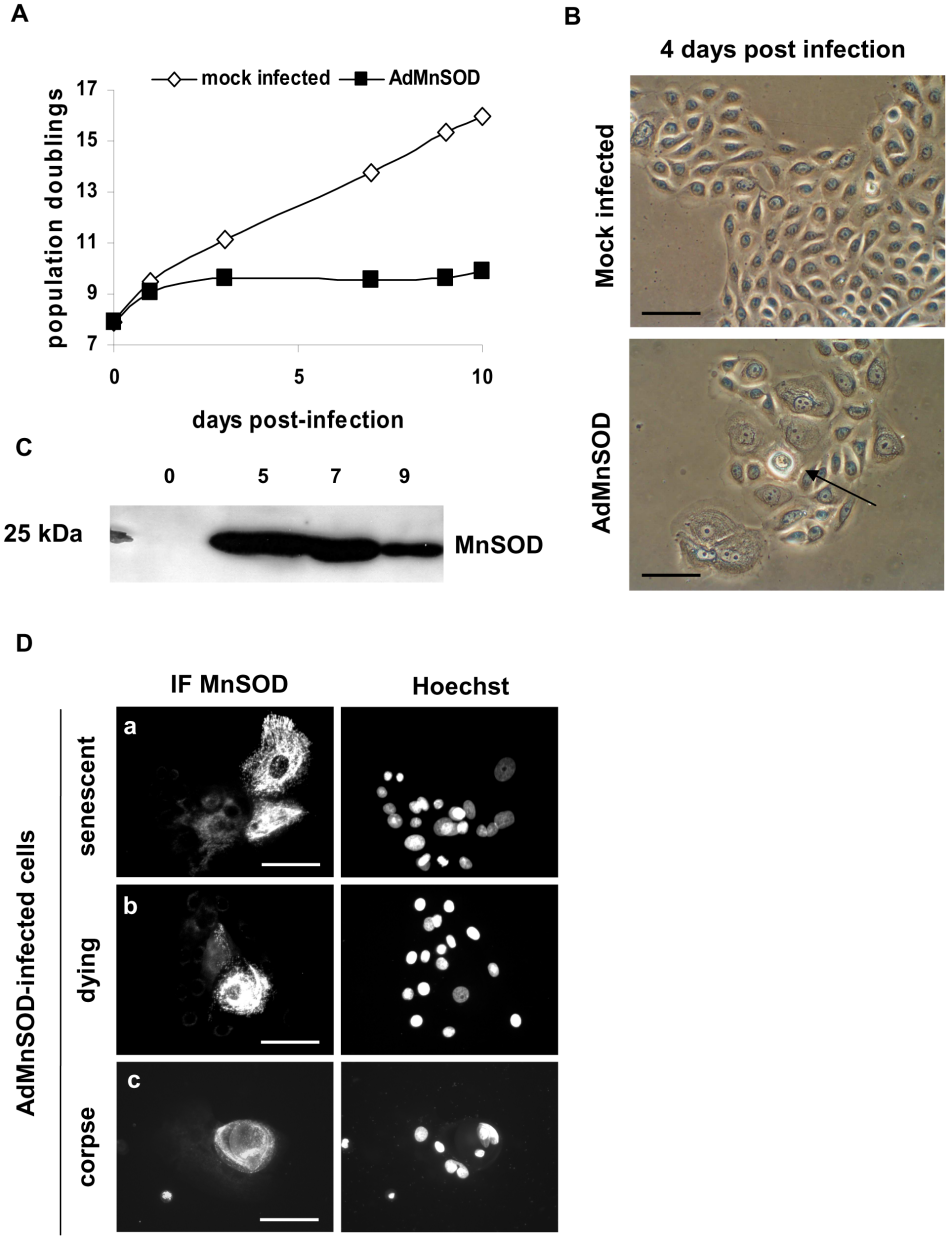
Overexpression of MnSOD induces premature senescence and autophagic cell death. NHEKs at the exponential growth phase were infected or not with an adenovirus encoding MnSOD (AdMnSOD) by directly adding virus particles in the culture medium at an input multiplicity of 200 viral particles/cell. (A) Growth curve of control and infected cultures. (B) Observation by phase contrast microscopy of the morphology of control and infected cells. Note that almost 100% of infected cells display a senescent-like morphology with spreading and polynucleation. One cell in the centre of the field resembles an autophagic corpse with a refringent central area (arrow). (C) Control of MnSOD expression in infected cells by western-blotting. (D) Immunofluorescence against MnSOD on AdMnSOD-infected cells. Amongst cells of an islet, cells expressing MnSOD at the highest level have either a marked senescent phenotype (a), a dying phenotype (b) or are already a corpse (c). Scale bars = 40 µM.

To assay the importance of this pathway in the induction of autophagic senescence-cell death, we overexpressed MnSOD in NHEKs at the exponential growth phase using an adenoviral vector (AdMnSOD), and we monitored the occurrence of senescence and autophagic cell death. Three days after infection, AdMnSOD-infected cells undergone a premature senescence growth plateau (Figure 6A), and from 4 days onwards, we observed cells with signs of autophagy, and corpses similar to those observed during normal senescence (Figure 6B). An immunofluorescence against MnSOD revealed that premature senescent cells and corpses express MnSOD at a very high level (Figure 6D). In corpses, most of the MnSOD staining was included into the central area (Figure 6D). These MnSOD-induced senescent cells and corpses contained a huge quantity of acidic vacuoles as indicated by a Lysotracker staining (Figure S2), intracellular Annexin-V staining (Figure S3A), 8-oxoG (Figure S3B) and AIP bridges (Figure S3C) similar to those of normal senescent NHEKs and corpses.

**Figure 7:**
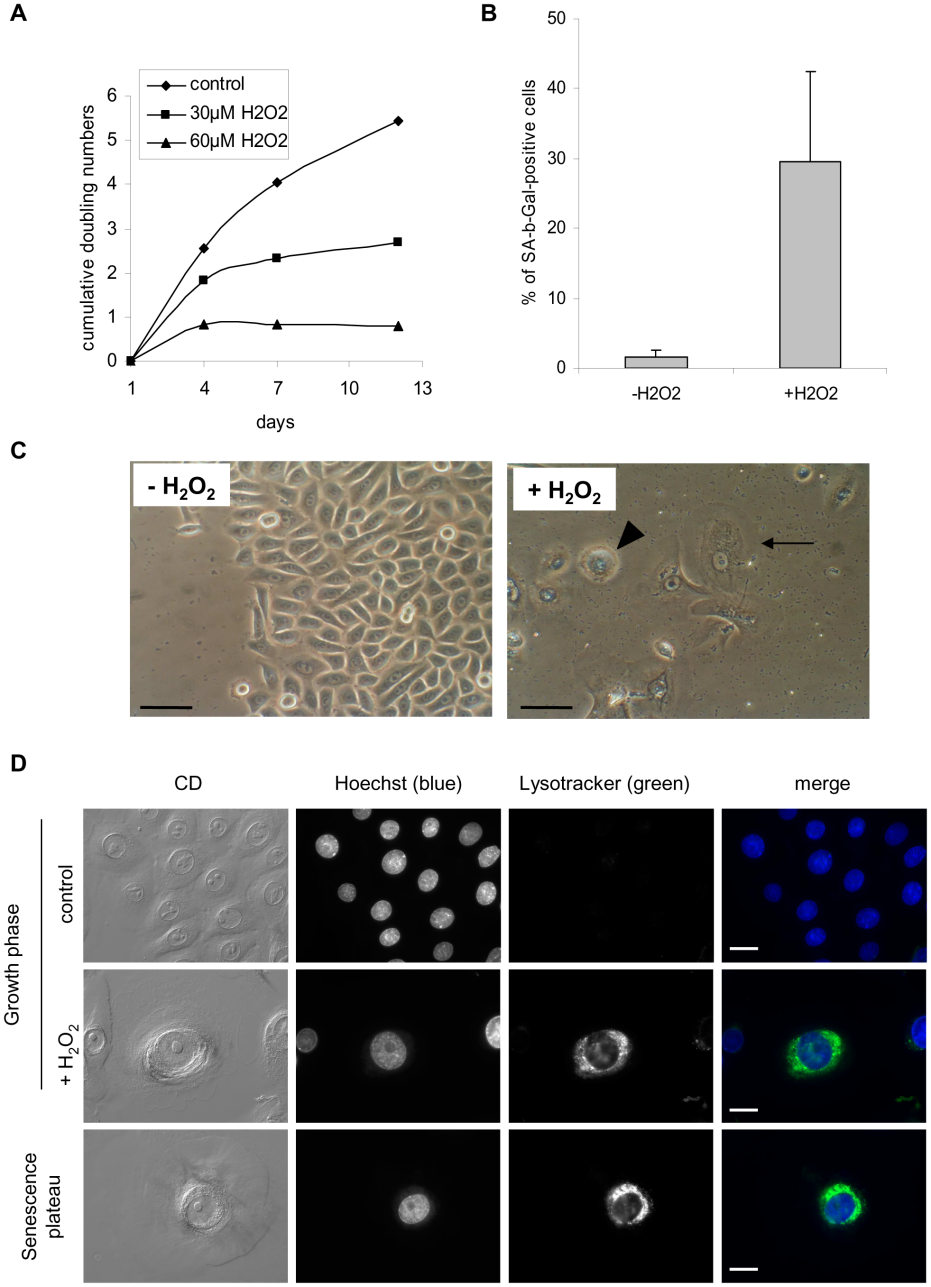
H_2_O_2_ induces premature senescence and signs of autophagic cell death. (A) NHEKs at the exponential growth phase were treated or not with 30 or 60 µM H_2_O_2_ for 2 hrs every 72 hrs. Cells were counted at different time points during the treatment in four independent wells, and the cumulative numbers of doublings were calculated using the mean of cell counts. (B) SA-beta-Gal assays were performed on control and 30 µM H_2_O_2_-treated cells at day 8. SA-beta-Gal-positive cells were counted in 4 microscopic fields. Results are given as means +/− SD of all field counts. Since the data are not strictly normally distributed, P values were calculated using both Student and Wilcoxon tests. They respectively equal 0.03 and 0.028. (C) Cells were observed under phase contrast microscopy. The images illustrate control and 30 µM H_2_O_2_-treated cell morphologies at day 8. Note the presence in H_2_O_2_-treated cultures of large senescent cells (arrow) and corpses (arrowhead). Scale bars = 20 µM. (D) NHEKs at the exponential growth phase were treated with 50 µM H_2_O_2_ and analyzed three days later by Lysotracker staining and microscopic analysis in comparison with cells at a normal senescence plateau. The corpses induced by the H_2_O_2_ treatment were similar to the normal ones occurring at the senescence plateau regarding their morphology under circular dichroism (CD) and their Lysotracker staining. The Lysotracker staining concentrates in the central area of corpses. Scale bars = 30 µM.

**Figure 8:**
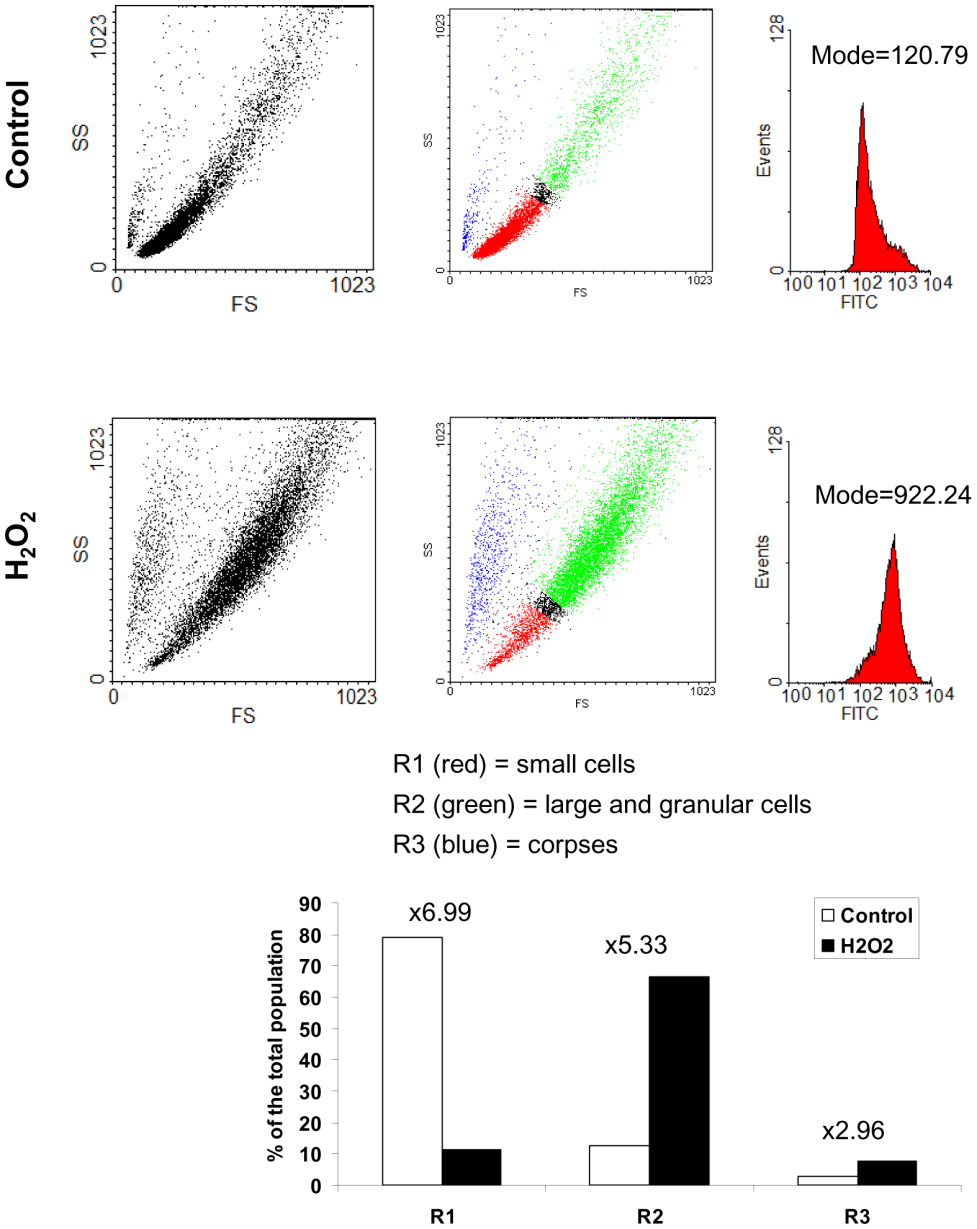
Quantification of the effect of the H_2_O_2_-treatement by flow cytometry and Lysotracker staining. NHEKs at the exponential growth phase were treated with 50 µM H_2_O_2_ and analyzed three days later by Lysotracker staining. (Left panel) Flow cytometry analysis of the cell population plot for forward factor (FS, indicative of size, in X) and side scatter factor (SS, indicative of granularity, in Y). (Middle panel) Quantitative analysis of the evolution of the subpopulations upon the H_2_O_2_ treatment using the WinMDI software. (Right panel) Measure of the intensity of the Lysotracker staining (FITC, in X) of the all population. The mode values of the fluorescence intensity are given.

**Figure 9:**
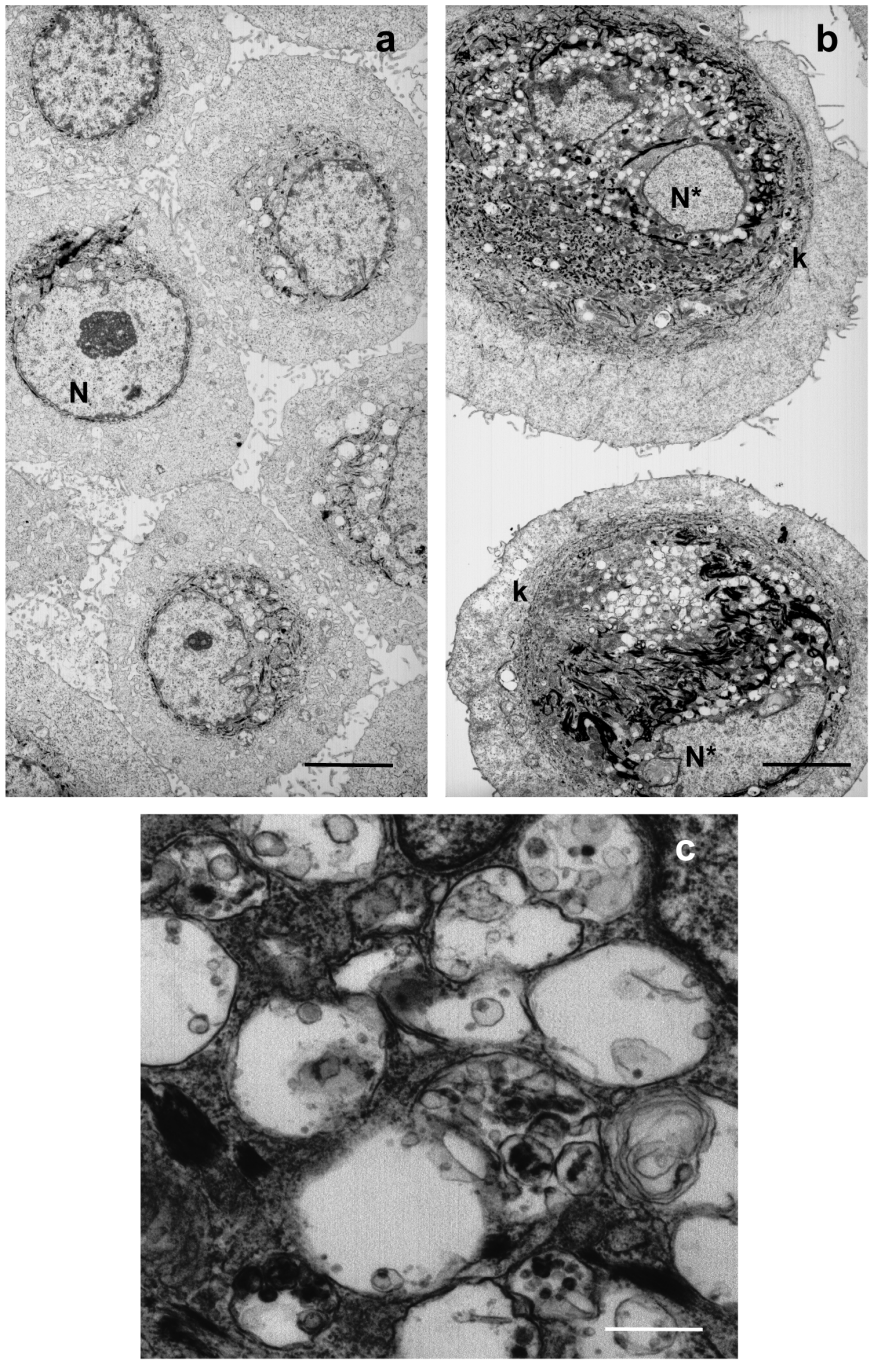
Ultrastructure of H_2_O_2_-induced senescent cells. NHEKs at the growth phase were treated with 50 µM H_2_O_2_ and processed 72 hrs later for transmission electron microscopy. (a) Control non treated cells. (b) H_2_O_2_-treated cells. N: nucleus, N*: deformed nucleus with less heterochromatin, k: cytokeratin network encircling the nucleus and the autophagic vacuoles. Scale bars = 5 µM. (c) Detail of autophagic vacuoles found in H_2_O_2_-treated cells. Scale bars = 0.4 µM.

**Figure 10:**
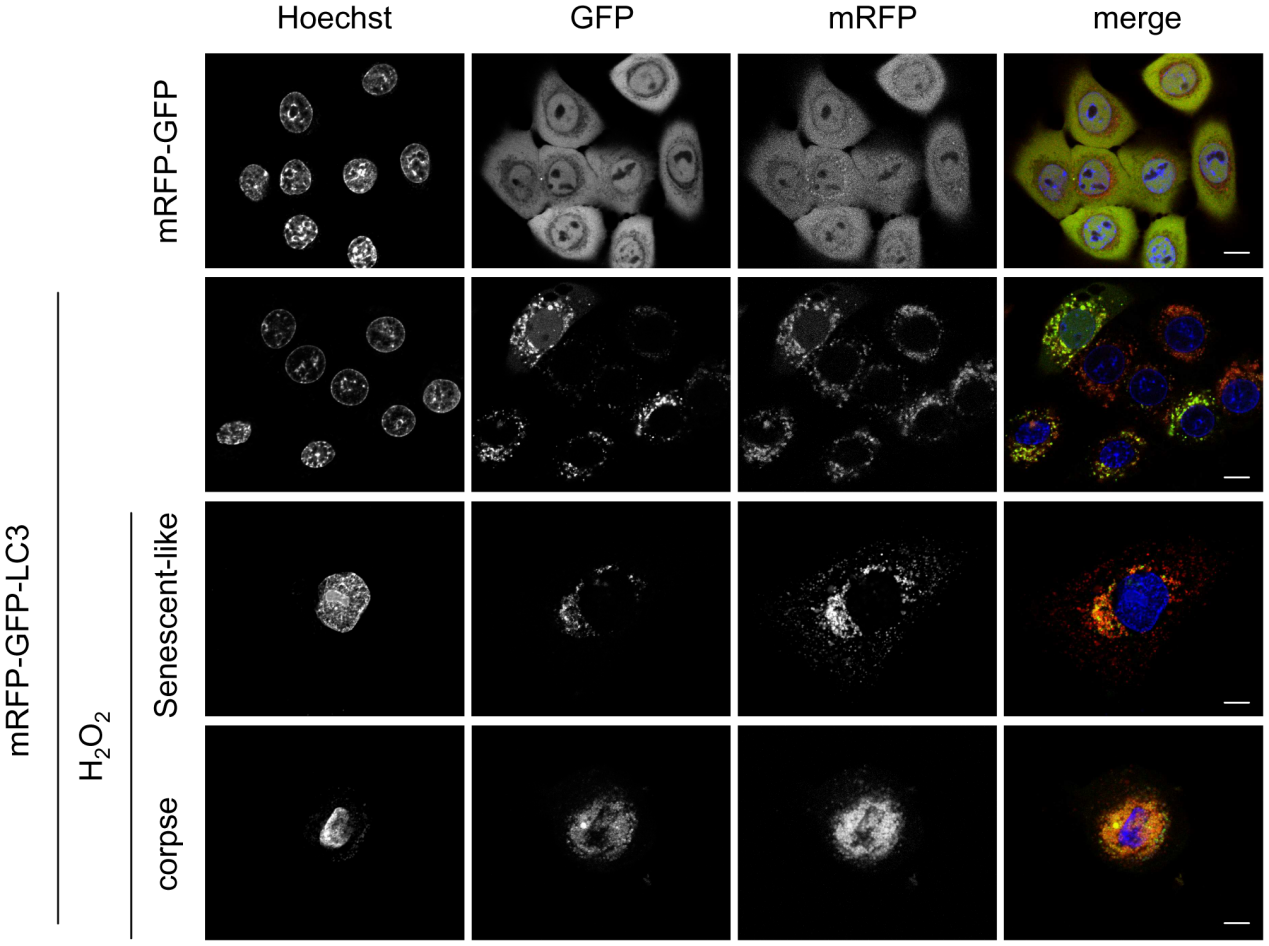
H_2_O_2_-induced senescent cells and corpses display a high and fully active autophagic flux. NHEKs at the exponential growth phase were treated or not twice with 50 µM H_2_O_2_ at 48 hrs interval and transfected with either the mRFP-GFP-LC3 or the control vector mRFP-GFP devoided of LC3. Forty eight hrs later, cells were analyzed under a confocal microscope. Non treated cells transfected with the control vector display a homogenous green (GFP) and red (mRFP) fluorescence. Non treated cells transfected by the mRFP-GFP-LC3 vector display some red and green vesicles. H_2_O_2_-treated cells transfected by the mRFP-GFP-LC3 vector display senescent or corpse features, with damaged nuclei and numerous vesicles whose majority are redder than green. In corpses, the vesicles are concentrated in the central area. Scale bars = 10 µM.

To confirm these results and to support a direct role of H_2_O_2_ in the induction of autophagic cell death of senescent cells, we directly treated NHEKs at the exponential growth phase with 30 to 60 µM H_2_O_2_. This treatment induced from 3 days onward a growth arrest (Figure 7A), the appearance of the SA-beta-Gal marker (Figure 7B), and phenotypes morphologically resembling senescence, with large and spread cells, signs of autophagy with numerous vacuoles and corpses with a refringent central area (Figure 7C). We compared the morphology of these H_2_O_2_-induced corpses with that of those normally occurring at the senescence plateau by performing an analysis by circular dichroïsm microscopy and Lysotracker staining. Corpses occuring in both conditions appeared completely similar, with the same load in and distribution of acidic vacuoles (Figure 7D). The quantification of the effects of the H_2_O_2_ treatment was made by flow cytometry. Cells were treated by 50 µM H_2_O_2_ and analyzed 3 days later according to their forward and side scatter values and their Lysotracker staining. We had previously shown that the subpopulation R1 with the smallest forward scatter and side scatter values corresponds to normal young living cells, the subpopulation R2, the largest and most granular, comprises cells with senescent features, and the subpopulation R3 with a small size but a high granularity corresponds to corpses with altered membranes [4]. The analysis shows that the H_2_O_2_ treatment increases the subpopulation of cells with senescent features about 5 fold, and the subpopulation of corpses about 3 fold (Figure 8). The analysis of the Lysotracker fluorescence intensity indicates an increase of about 7 fold of the mass of acidic vacuoles per cell (Figure 8). To make sure that the acidic vacuoles induced by the H_2_O_2_-treatment were autophagic vacuoles, we performed an analysis by transmission electron microscopy. This analysis confirmed the presence of numerous vacuoles in H_2_O_2_-treated cells, that appeared autophagic in nature because full of various membranous and non-membranous debris (Figure 9). We also used a mRFP-GFP tandem fluorescent-tagged Atg8/LC3 that enables to see the formation of autophagosomes by the vesiculation of the green and red fluorescent staining, and to see the ensuing maturation in autophagolysosomes by the keeping of the sole red fluorescent in some vesicles, the GFP being sensitive and the mRFP resistant to the acidic pH of autophagolysosomes [18]. Cells were treated twice with H_2_O_2_ at 48 hrs interval, transfected by the vector, and their fluorescence was analysed 48 hrs later. The results indicate that non treated cells display both green and red vesicles, and the H_2_O_2_-treated ones that have become senescent or dying display vesicles more numerous, aggregated in the vicinity of the nucleus, and numerous ones redder than green (Figure 10). Taken together, these results prove that a H_2_O_2_ treatment induces an intense and fully active autophagic flux, associated with senescence and cell death. We finally checked that H_2_O_2_-induced senescent cells and corpses do displayed oxidative damages similar to those observed in normal senescent cells and corpses, i.e. a membrane permeabilisation revealed by an intracellular Annexin-V staining (Figure S4), 8-oxoG (Figure S5A) and AIP bridges (Figure S5B).

Taken together, these results indicate that mimicking the MnSOD>H_2_O_2_ pathway that contributes to senescence in keratinocytes resumes senescence-associated oxidative damages and autophagic programmed cell death.

**Figure 11:**
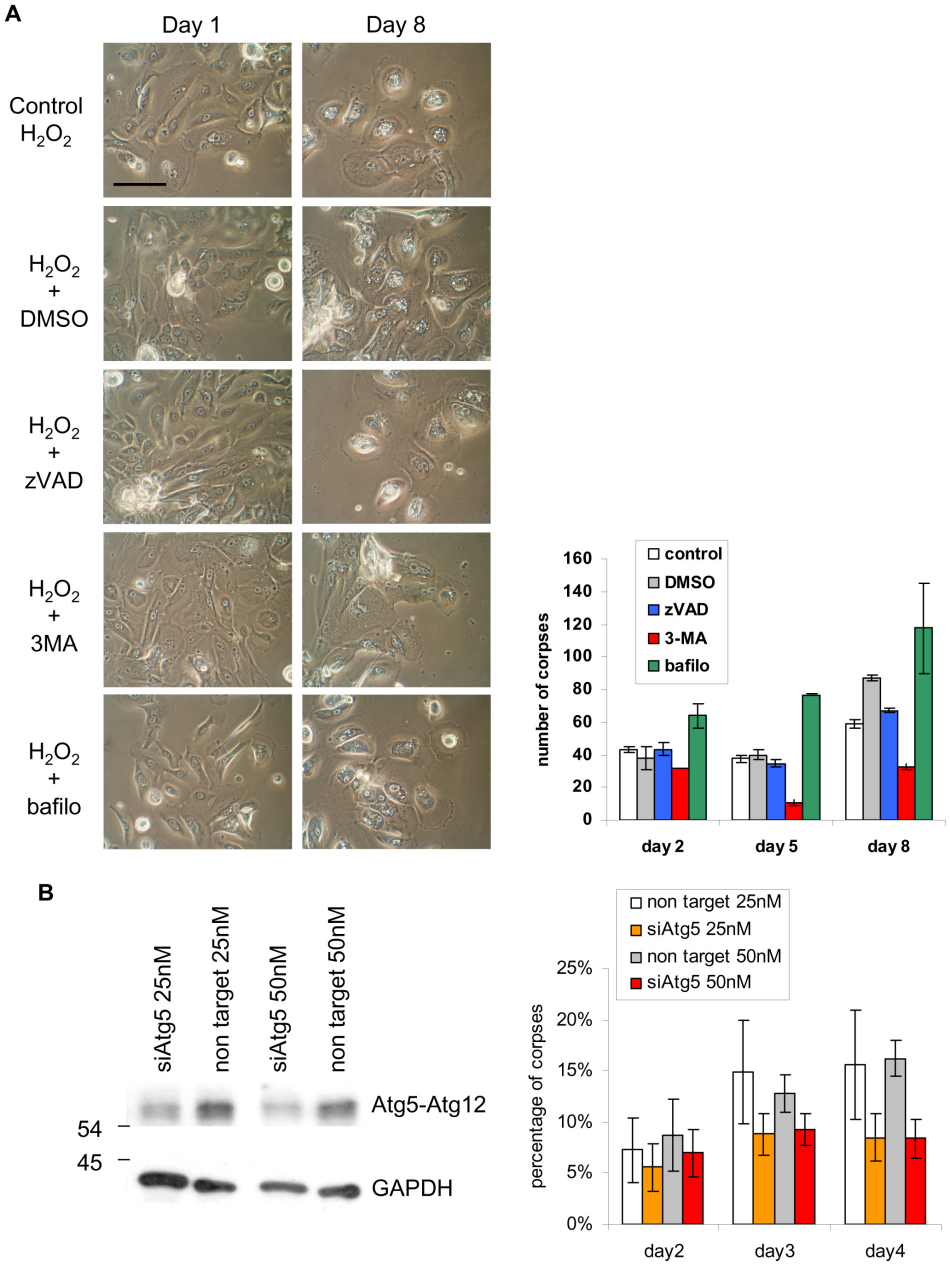
Inhibiting autophagy, but not apoptosis, delays the death of H_2_O_2_-induced senescent cells. (A) NHEKs at the exponential growth phase were treated by 50 µM H_2_O_2_ until almost 100% cells were senescent (after 48 hrs). Then, cells were either kept untreated for control, or treated by 3-MA at 5 mM, z-VAD at 20 µM, Bafilomycine A1 at 5 nM or DMSO, the diluent of z-VAD and Bafilomycine A1. Forty eight hours later, the H_2_O_2_-treatment followed by the inhibitor treatment was repeated a second time. (Upper panel) Cells morphologies observed under a phase-contrast microscope at different time points after the beginning of the inhibitor treatment. Scale bar = 50 µm. (Lower panel) The number of typical corpses with a refringent central area was counted under microscopic observation at the indicated time points after the beginning of the inhibitor treatment. The counts were performed in 20 microscopic fields of two independent culture dishes, each field comprising about 300 cells. The given results are the mean +/− standard deviation of all counts. Since the data are not strictly normally distributed, P values were calculated using both Student and Wilcoxon tests. The results are given Figure S6. This experiment is representative of two independent ones. (B) NHEKs at the exponential growth phase were treated twice by 50 µM H_2_O_2_ at 24 hrs interval until almost 100% cells were senescent (after 48 hrs). Then, cells were either transfected by anti-Atg5 siRNAs at 25 or 50 nM or by non target siRNAs at 25 or 50 nM. (Left panel) Western-blot analysis of Atg5 expression at day 4 after transfection. The antiAtg5 antibody recognizes the Atg5-Atg12 conjugate. (Right panel) The number of typical corpses with a refringent central area was counted under microscopic observation at the indicated time points after transfection. The counts were performed in 10 microscopic fields, each field comprising about 100 cells. The given results are the mean +/− standard deviation of all counts. Since the data are not strictly normally distributed, P values were calculated using both Student and Wilcoxon tests. The results are given Figure S6. This experiment is representative of three independent ones.

To definitely prove that the cell death following senescence induced by oxidative stress is driven by autophagy, we checked whether cell death level or time course could be affected by autophagy inhibitors. We used 3-methyladenine (3-MA), an inhibitor of the class III phosphatidylinositol 3-kinase (class III PI3K) complex involved in initial autophagosome formation [20], and Bafilomycin A1 an inhibitor of the H^+^ pump [21] that decreases the fusion of autophagosomes with lysosomes and the efficacy of digestion inside autophagolysosomes [22]. Because oxidative stress is also able to induce apoptosis, we also included in this assay zVAD, the specific caspase inhibitor. NHEKs at the exponential growth phase were treated by 50 µM H_2_O_2_ until almost 100% cells were senescent (after 48 hrs). Then, cells were either kept untreated or treated by 3-MA, Bafilomycin A1, z-VAD, or DMSO (the diluent of z-VAD and Bafilomycin A1). The treatments were repeated twice at 48 hrs intervals in order to enrich in corpses. Typical corpses with a refringent central area were counted under microscopic observation at the different time points. Their number increased with time in control cultures as well as in cultures treated with z-VAD. The number of corpses was significantly lower in cultures treated with 3-MA at 5 and 8 days of treatment (Figure 11A). These results suggest that NHEKs treated by 50 µM H_2_O_2_ die by autophagic programmed cell death, not by apoptosis. In cultures treated with Bafilomycin A1, we observed an accumulation of corpses with a very refringent central area (Figure 11A). We have previously demonstrated by transmission electron microscopy that normal senescent cultures treated by Bafilomycin A1 evolve in corpses completely congested by numerous autophagic vacuoles, themselves full of various components incompletely degraded [4]. This accumulation of non degraded material inside autophagic vacuoles in corpses upon Bafilomycin A1 treatment confirms that the autophagic flux is active during NHEK autophagic death. Since pharmacological inhibitors are not completely specific, we performed a supplementary experiment using siRNA directed against Atg5, a protein involved in the lipidation of Atg8/LC3 [23] and hence in autophagosome formation. Cells were treated twice with 50 µM H_2_O_2_ at 48 hrs interval to induce premature senescence, and 24 hrs later transfected with anti-Atg5 siRNAs or non target siRNAs. The efficacy of siRNA was checked by western-blotting at day 4 after transfection, and the accumulation of corpses was quantified by counting under microscopic observation. Four days after transfection, corpses were about two fold less numerous in cells transfected by anti-Atg5 siRNAs than in cells transfected by non target siRNAs (Figure 11B), hence confirming that cell death following H_2_O_2_-induced premature senescence occurs through autophagy.

### Lowering oxidative stress decreases the autophagic activity and the death of senescent keratinocytes

**Figure 12:**
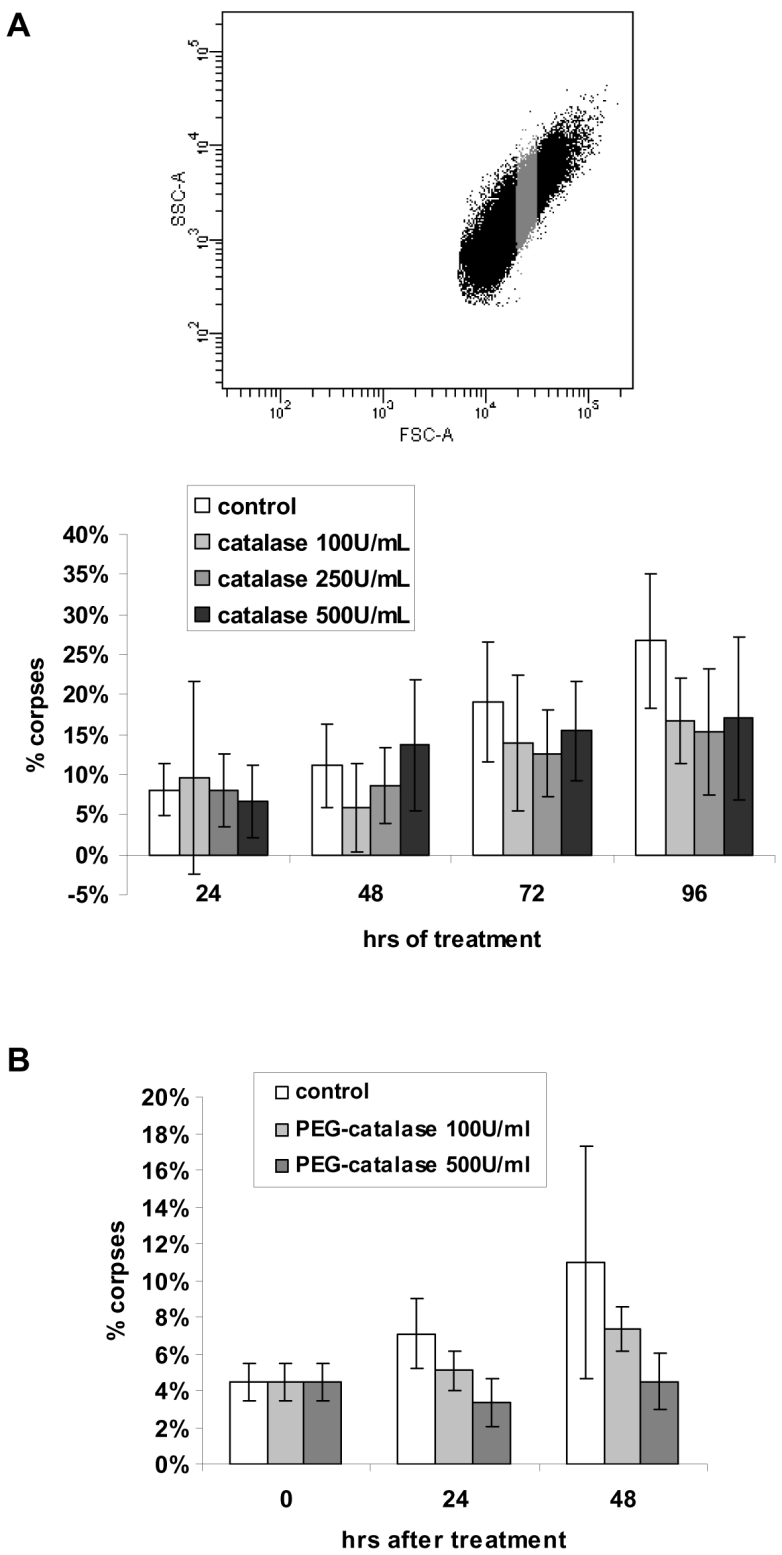
Enhancing H_2_O_2_ degradation delays senescent-cell death. (A) NHEKs at the senescence growth plateau were analyzed by flow cytometry according to size (FSC in X) and granularity (SSC in Y) and the subpopulation of still viable senescent cells (in grey) was sorted. Sorted cells were plated in 6-wells plates at 30.000 cells per well and treated or not by catalase at different concentrations. The medium +/− catalase was renewed every 24 hrs. The number of typical corpses with a refringent central area was counted under microscopic observation every day. The counts were performed in 12 microscopic fields in three independent culture wells, each field comprising about 10 cells. The given results are the mean +/− standard deviation of all counts. Since the data are not strictly normally distributed, P values were calculated using both Student and Wilcoxon tests. The results are given Figure S6. This experiment is representative of two independent ones. (B) NHEKs at the senescence growth plateau were plated in 6-wells plates at 500.000 cells per well and treated or not by PEG-catalase at different concentrations. The number of typical corpses with a refringent central area was counted under microscopic observation every day. The counts were performed in 6 microscopic fields comprising about 100 cells. The given results are the mean +/− standard deviation of all counts. Since the data are not strictly normally distributed, P values were calculated using both Student and Wilcoxon tests. The results are given Figure S6. This experiment is representative of two independent ones.

Finally, to definitely prove that autophagic cell death is activated in senescent cells in response to oxidative damage, we examined whether antioxidants could decrease senescent-cell death rate or kinetics. As antioxidant, we used catalase which specifically degrades H_2_O_2_ into H_2_O. We used either native catalase since we previously showed that it is able to delay the NHEK senescence plateau [10] or PEG-catalase, a monomethoxy-polyethylene glycol conjugated catalase which is more efficient because more resistant to protease attacks [24] and able to bind plasma membranes [25]. In a first series of experiments, native catalase was applied to a subpopulation of still alive senescent cells sorted by FACS [4] (Figure 12A) every 24 hrs, during 4 days, and corpses were counted under a phase contrast microscope. The percentage of corpses in the non treated cell population increased about 3.33 fold in 4 days, whereas it increased only about 1.9 fold in the catalase-treated cell population, hence representing a 42.9% inhibition of senescent-cell death (Figure 12A). In a second series of experiments, PEG-catalase was applied to a total population of NHEKs at the senescent plateau, and corpses were counted after 24 and 48 hrs. With that protocol, the inhibition of senescent-cell death was more efficient (58.7% inhibition) and more rapid (Figure 12B). Both these experiments demonstrate that senescent-cell death is induced consequently to H_2_O_2_ accumulation.

## Discussion

### Autophagy as a cell death mechanism in senescent keratinocytes

Macroautophagy is a process involved in proteins and organelles normal turnover; it is referred as such as constitutive autophagy. It is also a survival process induced to resist to nutrient deprivation, referred as starvation-induced autophagy [9]. However, macroautophagy is also a cell death process, referred as type II programmed cell death [26]. This type of cell death was described in several model organisms and seems to involve the same set of Atg proteins as those involved in constitutive and starvation-induced autophagy [27], [28]. Therefore, how autophagy can shift from a survival process to a lethal one is not entirely clear. Several parameters seem important: the level of the autophagic activity that have to be high enough to destroy major portions of cytosol and organelles [29], the levels of expression of Atg5 and Atg6/Beclin-1 that do not change during starvation-induced autophagy but increase during autophagic cell death [30], and the possible selective degradation of some vital components or proteins, such as catalase, a major antioxidant enzyme [31]. We and others have demonstrated in recent works that autophagy is activated in normal or RAS-induced senescent cells, and that the autophagic flux is fully functional and contribute to the establishment of the senescent phenotype [4], [5]. In addition, we have shown in keratinocytes that this autophagic activity is high enough to end up in the death of senescent cells. Notably, we have evidenced in normal senescent keratinocytes an increase in Atg6/Beclin-1, and an accumulation of a huge quantity of autophagic vacuoles [4]. We have not observed any increase in catalase degradation, either in the total population at the senescent plateau [10], or in senescent cells and corpses sorted by FACS (data not shown).

### Oxidative stress as an inducer of autophagic cell death in senescent keratinocytes

Several data have suggested that the damaged cell components that are targeted for autophagy are those that are oxidized [32]. Moreover, diverse inducers of autophagic programmed cell death were shown to act through the generation of reactive oxygen species (ROS) [33], more specifically H_2_O_2_ which was shown to act as a signalling molecule able to activate the initial autophagosome formation, via the oxidation of a specific cystein of Atg4 [34]. We have demonstrated in a previous study that H_2_O_2_ is accumulated in senescent keratinocytes following the activation of NF-kappaB and the upregulation of MnSOD, and contributes to the occurrence of the senescent phenotype [10]. We complete here these data by showing that this oxidative stress pathway is also at the origin of a high autophagic activity that becomes fatal to senescent keratinocytes. Indeed, we show that mimicking this pathway by overexpressing MnSOD or directly adding H_2_O_2_ to cultures of young keratinocytes induced a premature senescence plateau followed by autophagic cell death; the level of this ROS-induced autophagic cell death is decreased in the presence of 3-MA or anti-Atg5 siRNAs. Conversely, treating senescent cells with the H_2_O_2_-degrading enzyme catalase reduced their level of autophagic cell death.

MnSOD being a mitochondrial enzyme, the first targets of the H_2_O_2_ it produces should be mitochondria, potentially explaining why they are swelled and aggregated in advanced senescent cells. A surprising point is the increase in mitochondria number during senescence despite their damaging. Such an increase was already documented in MRC5 fibroblasts following H_2_O_2_-induced premature senescence, suggesting that mitochondrial biogenesis is unaffected by oxidative damage [35]. Besides mitochondria, H_2_O_2_ can also affect nuclei. Indeed, being diffusible across membranes [36], H_2_O_2_ could easily reach the nucleus, all the more because damaged mitochondria are agglutinated in its vicinity. The presence of 8oxoG and AIP bridges on mitochondria and nuclei inside the central area of corpses where autophagic vesicles accumulate argues that mitochondria and nuclei of senescent cells are targeted to autophagic elimination because of their oxidative damages. Such an elimination of oxidized mitochondria was already documented in several models [37]–[39]. The possible elimination of nuclei by autophagy because of their oxidative damage is suggested by several studies: it was shown that H_2_O_2_-induced DNA breaks can induce autophagy through the activation of PARP-1 [40]; The inhibition of DNA-PK, a nuclear kinase involved in DNA break signalisation, was shown to sensitize to autophagy [41]; Treatments with an other DNA damaging agent, the etoposide, were shown to induce autophagy and senescence [5] and to result in autophagic programmed cell death through the increase in Beclin-1 [30]. Moreover, DNA damage has been shown to accumulate in cancer cells deficient in autophagy [42]. Despite this evidence of an autophagy-inducing activity of DNA damages, the direct autophagic elimination of nuclei by autophagy is up to now poorly documented. In yeasts, nuclei could be degraded by a process morphologically resembling microautophagy [43] but involving the core macroautophagy genes [44]. In mammalian cells, one study has illustrated by electron microscopy the possible nucleus encircling by a phagocytic double membrane [45]. Recently, another study gave evidence of nuclear components degradation by perinuclear giant autophagic vacuoles [46].

Therefore, we propose the following scenario: with time and doublings, senescent keratinocytes become cell-cycle arrested and accumulate ROS, especially H_2_O_2_, in part through the activation of NF-kappaB and the upregulation of MnSOD [10]. Oxidatively-damaged mitochondria loss their attachment to microtubules, hence aggregate in the vicinity of the nucleus, which favours its oxidative attack. In consequence to these oxidative damages, and potentially through the oxidation of Atg4 by H_2_O_2_, autophagy is highly activated and progressively eliminates all the oxidized vital cell components, leading to cell death.

### Apoptosis versus autophagic cell death in senescent cells

In a previous work, we had demonstrated that normal senescent keratinocytes do not die by apoptosis [4]. Here, we add that H_2_O_2_-induced senescent-like keratinocytes do not die by apoptosis as well. However, oxidative stress is a well known apoptosis inducer. Actually, we observed an increase in apoptotic cells from 1–2% in the population of exponentially growing keratinocytes to 5–8% at the senescence plateau (data not shown). In parallel, we observed that H_2_O_2_ treatments induce different outcomes for keratinocytes according to the concentration used: as shown in this paper, low concentrations of H_2_O_2_ induced in a few days a senescent-like phenotype followed by autophagic programmed cell death, but higher concentrations induced in a few hours an apoptotic cell death characterized by typical membranous Annexin-V staining and cytoplasm/nuclear condensation (data not shown). A study comparing senescent endothelial HUVECs with senescent fibroblasts showed that senescent HUVECs display many signs of apoptosis, whereas senescent fibroblasts do not [47]. The authors associated the death of senescent HUVECs by apoptosis with the generation of oxidative stress during senescence [48] and proposed that senescent fibroblasts do not die by apoptosis because they are more resistant to oxidative stress than HUVECs [49]. Therefore, apoptosis is likely to co-exist with autophagic programmed cell death during senescence, but in low proportions, and only in the case of high level of oxidative stress or especially sensitive cell-types.

### Implications in aging

Do old cells die through autophagic programmed cell death *in vivo* during aging as in culture because of oxidative damages? Probably yes. Indeed, the universal marker of senescence, the SA-beta-Gal activity [14], is actually an indirect marker of autophagy since it reflects the activity of a lysosomal enzyme, and hence the mass of lysosomes [50]. The number of cells positive for this marker was shown to increase during normal human and mouse aging [14], [51]–[53], suggesting that the autophagic activity increases in cells during aging. Lipofuscin, the well-known marker of aged skin and other organs, is an aggregate of proteins having reacted with lipid peroxidation end-products that accumulates with advancing age inside autophagic vacuoles [54]. Hence, it is a marker of autophagy resulting from oxidative damage. Consequently, its accumulation in cells during aging not only confirms that the autophagic activity increases with age, but in addition that it does in response to oxidative damage.

### Implications for tumorigenesis

An important consequence of our understanding of the role of oxidative stress in senescent-cell death concerns the relationship between aging and cancer. Indeed, there are numerous evidences that autophagy is down-regulated in cancer cells. Beclin-1 is often mono-allelically deleted in various carcinomas [29] and its heterozygous disruption in mice caused spontaneous tumours [55], [56]. The tumour suppressor PTEN, that rivals p53 in being the most frequently mutated gene in human cancer [57], promotes autophagy in HT-29 colon cancer cells by blocking the Akt survival pathway. Mutations in PTEN result in inactivation of autophagy and tumour formation [58]. Therefore, one can speculate that during senescence, most oxidatively-altered cells would die by autophagy, but some cells would beneficiate from the mutagenicity of ROS to escape autophagic cell death and evolve in transformed long-lived cells. Hence, acquiring autophagic cell death resistance would be an event as important as becoming apoptosis resistant for neoplastic transformation. However, this autophagic resistance should be only partial so that, once formed, cancer cells can normally use constitutive autophagy for turnover ensuring, or have recourse to starvation-induced autophagy in case of limited angiogenesis.

## Supporting Information

Figure S1 Senescent keratinocytes accumulate reactive oxygen species NHEKs at the exponential growth phase or at the senescence plateau were suspended and stained with H2-DCFDA. They were then analyzed by flow cytometry for forward (FS, indicative of size, in Y in the dot plot) and side scatter (SS, indicative of granularity, in X in the dot plot) factors and H2-DCFDA fluorescence intensity (GFP on the histograms). The senescent population increases in size and granularity; its H2-DCFDA increases about ten fold.
(0.31 MB TIF)

Figure S2 Senescent cells and corpses induced by MnSOD overexpression have an increased number of acidic organelles NHEKs at the exponential growth phase were infected with AdMnSOD as in Fig. 6 and 4 days later they were stained with Lysotracker as in Fig. 3. Note that in AdMnSOD-infected cultures, cells with a marked senescent phenotype (green arrows) and corpses (red arrows) display a high Lysotracker staining. Scale bars = 40 µM.
(1.84 MB TIF)

Figure S3 Corpses induced by MnSOD overexpression have their membranes altered and display oxidative damages NHEKs at the exponential growth phase were infected with AdMnSOD as in Fig. 6. (A) Annexin-V assays performed 10 days post-infection. Both typical large senescent cells and corpses that appeared in the AdMnSOD-infected cultures display some staining of their endomembranes. Scale bars = 30 µM. (B) Immunodetection of 8-oxo-guanines (8oxoG) 5 days post-infection. The image shows an example of the high staining of a corpse and of a senescent cell (C) Immunodetection of amino-imino-propene (AIP) bridges 10 days post-infection. The image shows an example of the high staining of a corpse. Scale bars = 40 µM.
(2.63 MB TIF)

Figure S4 H2O2-induced senescent cells and corpses have permeabilized membranes NHEKs at the exponential growth phase were treated with H2O2 as in Fig. 7 and processed for Annexin-V assay. H2O2-treated cells with senescent morphology and corpses display intracellular staining, revealing that their membranes are permeabilized. Scale bars = 40 µM.
(2.02 MB TIF)

Figure S5 H2O2-induced senescent cells and corpses display oxidative damages NHEKs at the exponential growth phase were treated with 50 µM H2O2 and processed 48 hrs later for immunofluorescence against 8-oxo-guanines (8oxoG) and amino-imino-propene (AIP) bridges. Nuclei were counterstained with Hoechst. Cells were observed under epifluorescence microscopy and circular dichroism (CD). (A) 8oxoG staining. Both cells with a senescent-like morphology and corpses display some staining of cytoplasmic structures and some staining inside the nucleus. In corpses, the cytoplasmic staining is concentrated in the central area. (B) AIP bridges staining. Cells with a senescent-like morphology display a nuclear staining and some diffuse cytoplasmic staining. In corpses, the nuclear staining is very intense, and the cytoplasmic staining is concentrated in the central area. Scale bars = 20 µM.
(2.93 MB TIF)

Figure S6 Statistical analysis of the results of Figures 11 and 12.
(0.61 MB TIF)
