# Supplementary figures and images for: MnSOD Upregulation Induces Autophagic Programmed Cell Death in Senescent Keratinocytes
Source: PLoS One. 2010 Sep 14;5(9):e12712. doi: 10.1371/journal.pone.0012712 (PMC2939051; doi:10.1371/journal.pone.0012712)

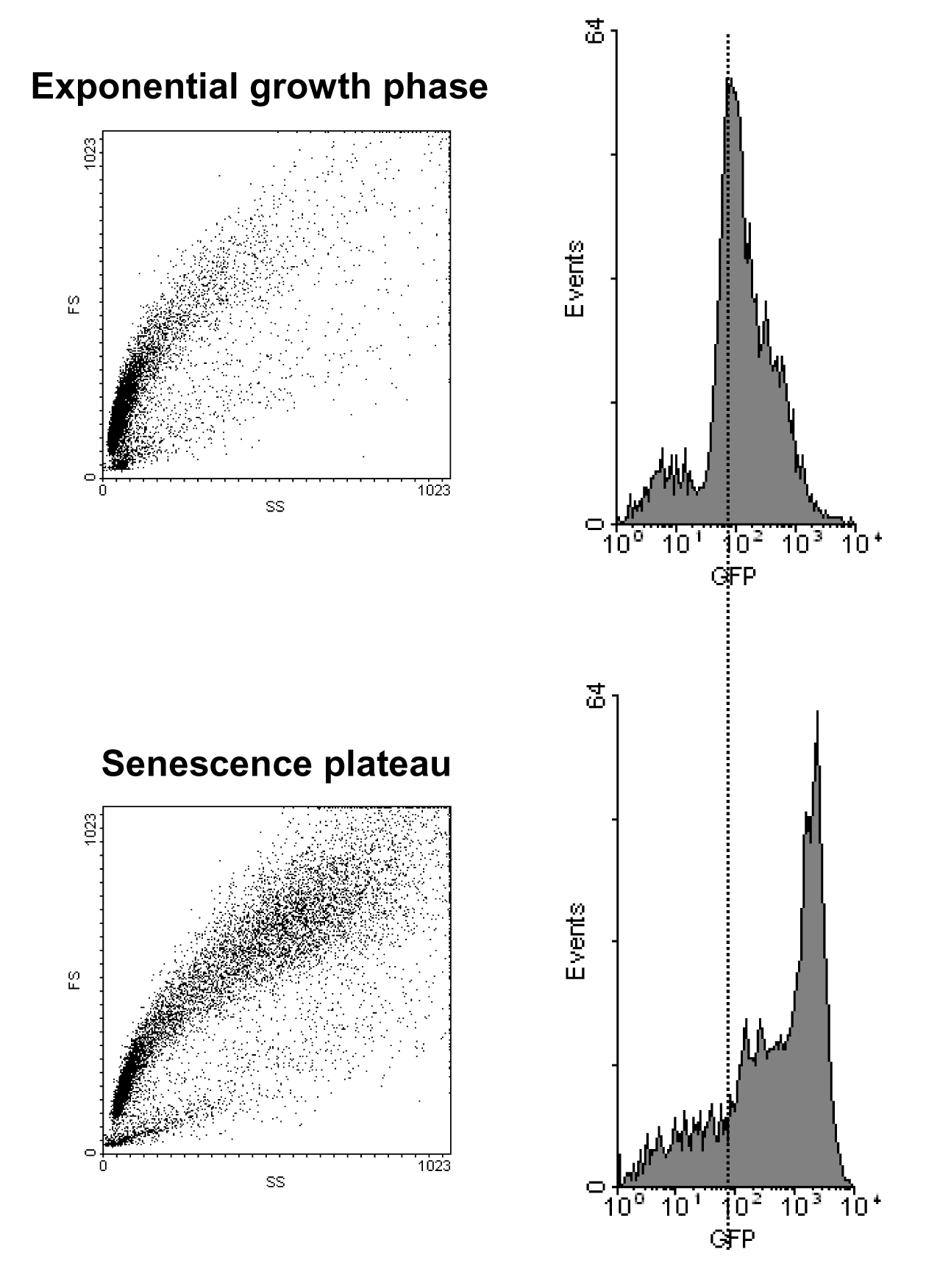

Supplement: Figure S1 — Senescent keratinocytes accumulate reactive oxygen species NHEKs at the exponential growth phase or at the senescence plateau were suspended and stained with H2-DCFDA. They were then analyzed by flow cytometry for forward (FS, indicative of size, in Y in the dot plot) and side scatter (SS, indicative of granularity, in X in the dot plot) factors and H2-DCFDA fluorescence intensity (GFP on the histograms). The senescent population increases in size and granularity; its H2-DCFDA increases about ten fold. (0.31 MB TIF) [file pone.0012712.s001.tif]

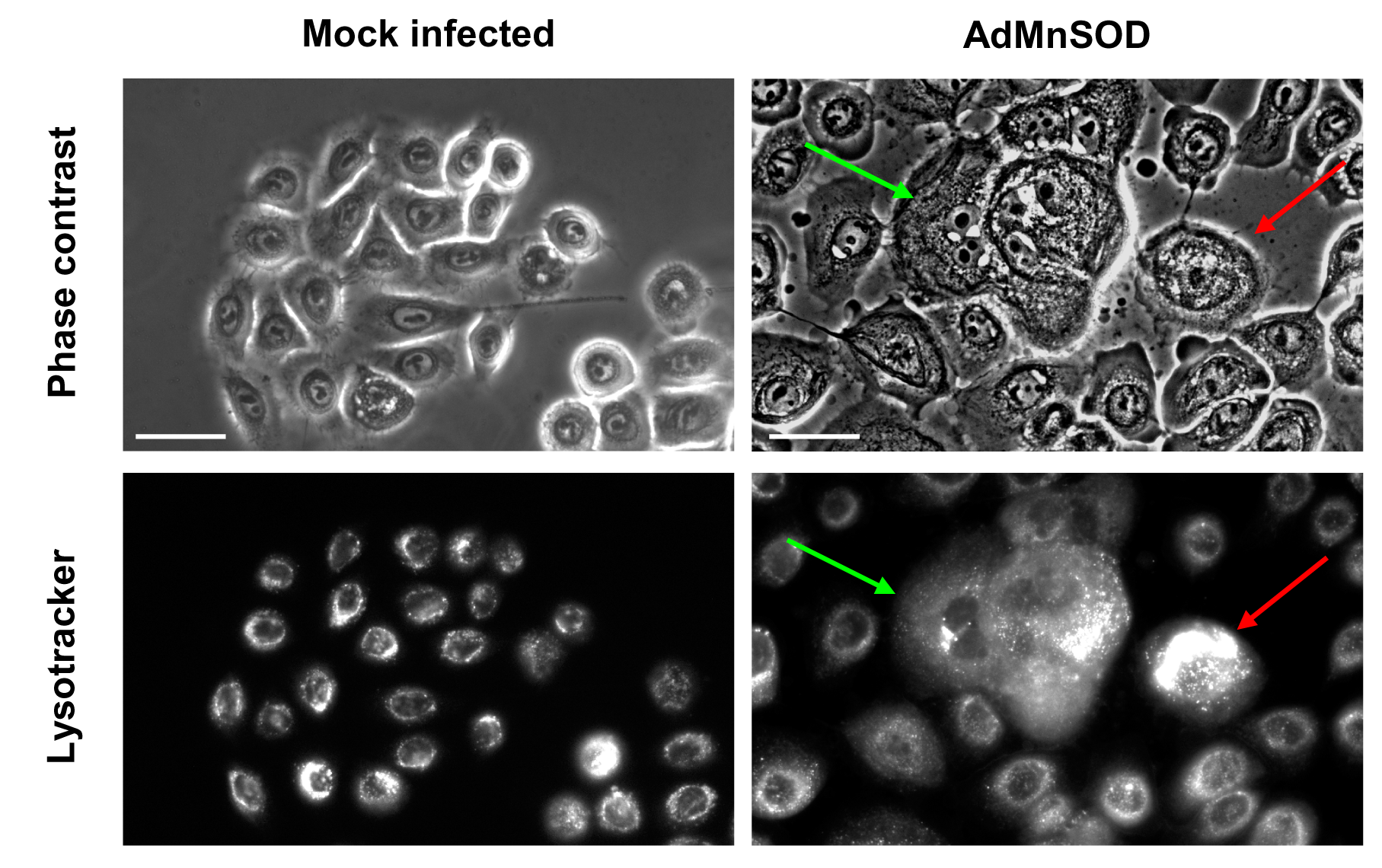

Supplement: Figure S2 — Senescent cells and corpses induced by MnSOD overexpression have an increased number of acidic organelles NHEKs at the exponential growth phase were infected with AdMnSOD as in Fig. 6 and 4 days later they were stained with Lysotracker as in Fig. 3. Note that in AdMnSOD-infected cultures, cells with a marked senescent phenotype (green arrows) and corpses (red arrows) display a high Lysotracker staining. Scale bars = 40 µM. (1.84 MB TIF) [file pone.0012712.s002.tif]

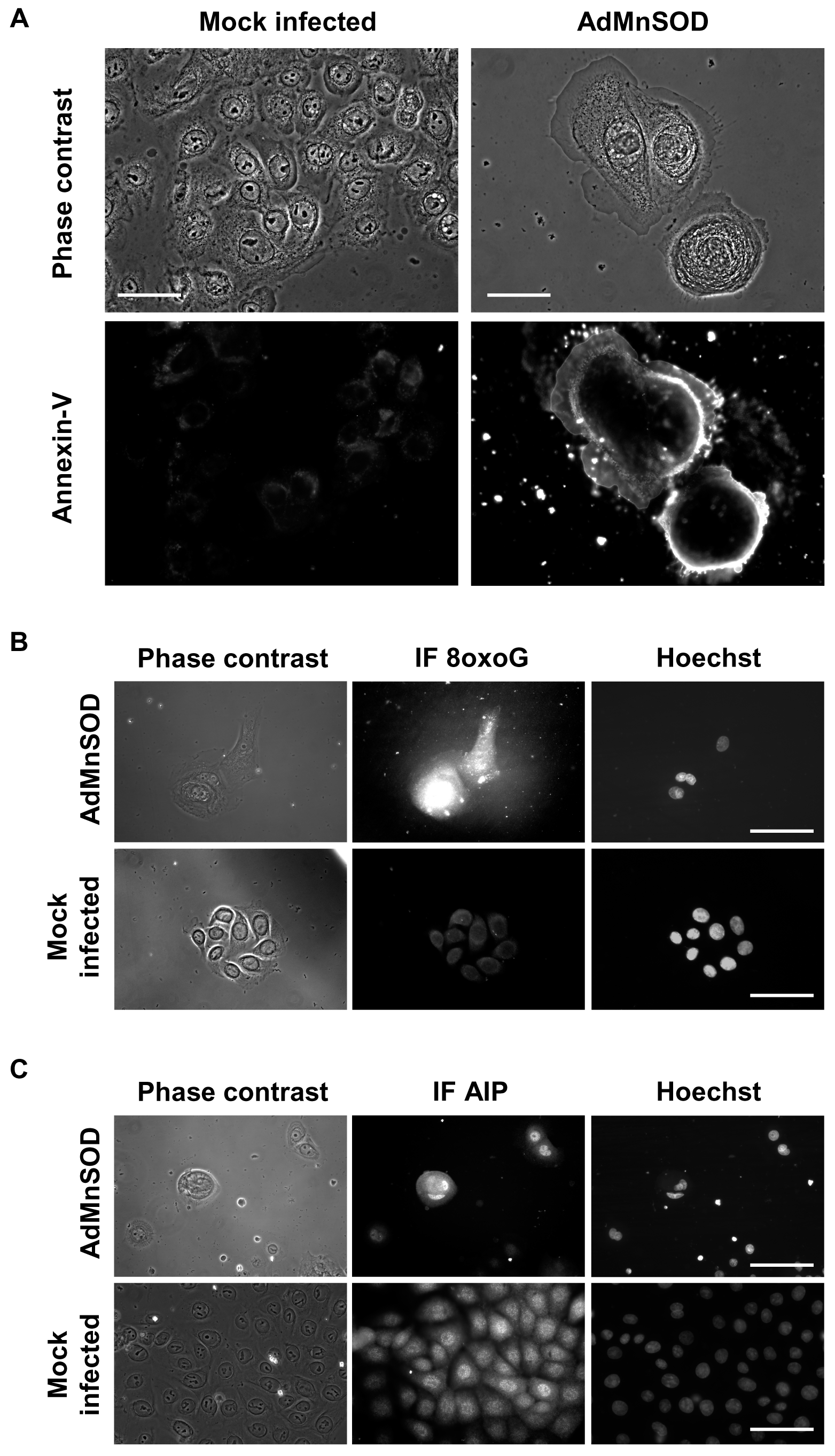

Supplement: Figure S3 — Corpses induced by MnSOD overexpression have their membranes altered and display oxidative damages NHEKs at the exponential growth phase were infected with AdMnSOD as in Fig. 6. (A) Annexin-V assays performed 10 days post-infection. Both typical large senescent cells and corpses that appeared in the AdMnSOD-infected cultures display some staining of their endomembranes. Scale bars = 30 µM. (B) Immunodetection of 8-oxo-guanines (8oxoG) 5 days post-infection. The image shows an example of the high staining of a corpse and of a senescent cell (C) Immunodetection of amino-imino-propene (AIP) bridges 10 days post-infection. The image shows an example of the high staining of a corpse. Scale bars = 40 µM. (2.63 MB TIF) [file pone.0012712.s003.tif]

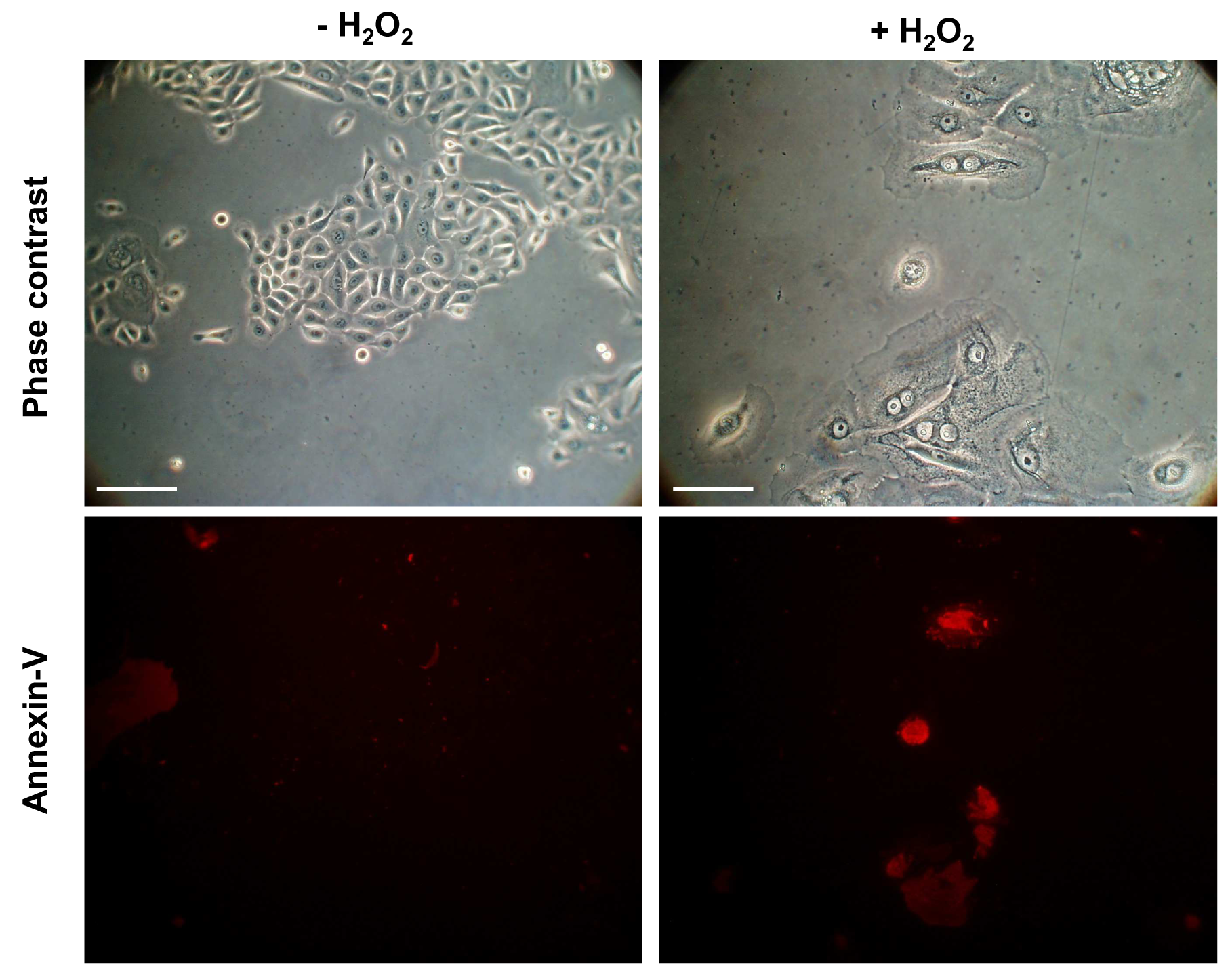

Supplement: Figure S4 — H2O2-induced senescent cells and corpses have permeabilized membranes NHEKs at the exponential growth phase were treated with H2O2 as in Fig. 7 and processed for Annexin-V assay. H2O2-treated cells with senescent morphology and corpses display intracellular staining, revealing that their membranes are permeabilized. Scale bars = 40 µM. (2.02 MB TIF) [file pone.0012712.s004.tif]

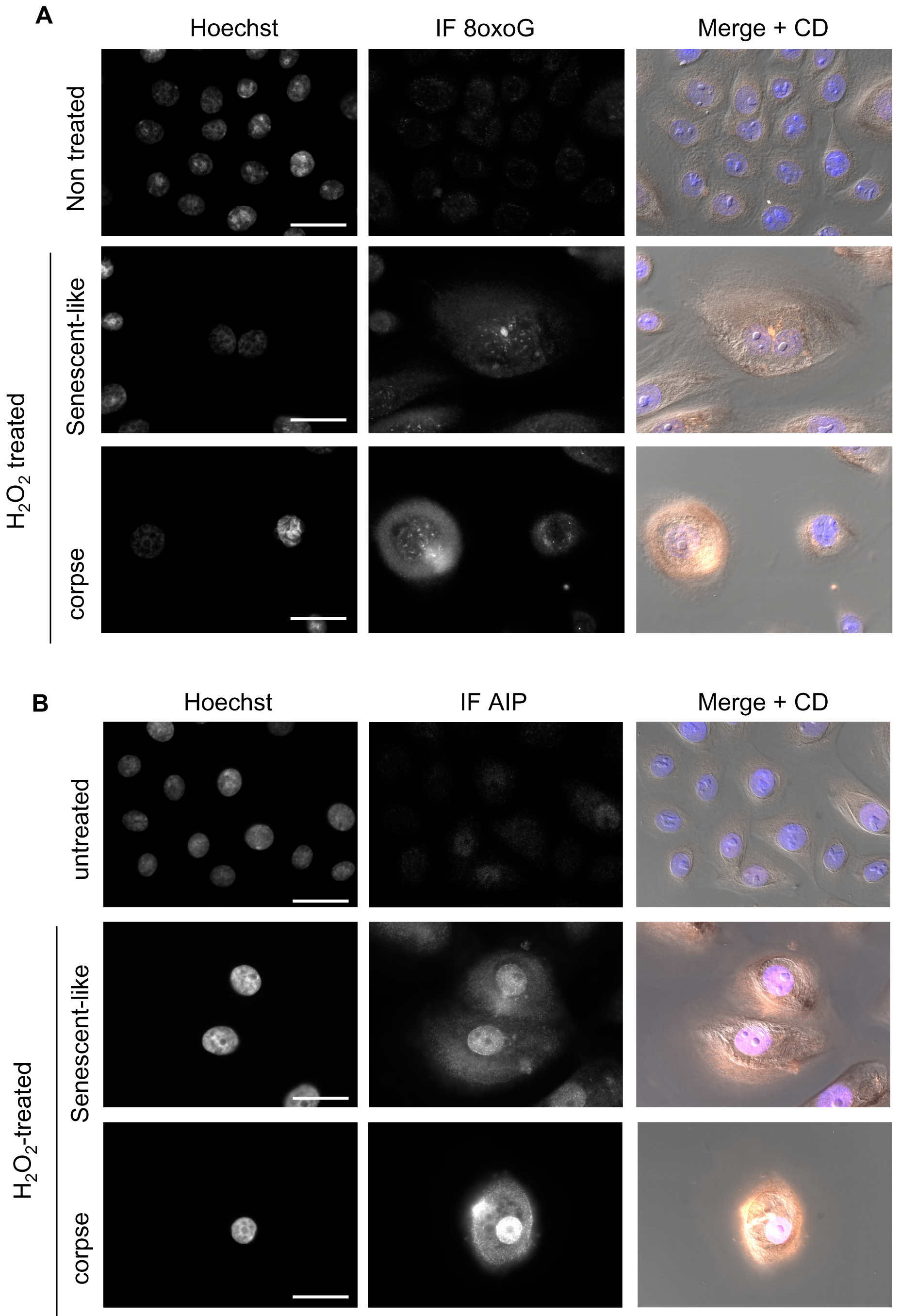

Supplement: Figure S5 — H2O2-induced senescent cells and corpses display oxidative damages NHEKs at the exponential growth phase were treated with 50 µM H2O2 and processed 48 hrs later for immunofluorescence against 8-oxo-guanines (8oxoG) and amino-imino-propene (AIP) bridges. Nuclei were counterstained with Hoechst. Cells were observed under epifluorescence microscopy and circular dichroism (CD). (A) 8oxoG staining. Both cells with a senescent-like morphology and corpses display some staining of cytoplasmic structures and some staining inside the nucleus. In corpses, the cytoplasmic staining is concentrated in the central area. (B) AIP bridges staining. Cells with a senescent-like morphology display a nuclear staining and some diffuse cytoplasmic staining. In corpses, the nuclear staining is very intense, and the cytoplasmic staining is concentrated in the central area. Scale bars = 20 µM. (2.93 MB TIF) [file pone.0012712.s005.tif]

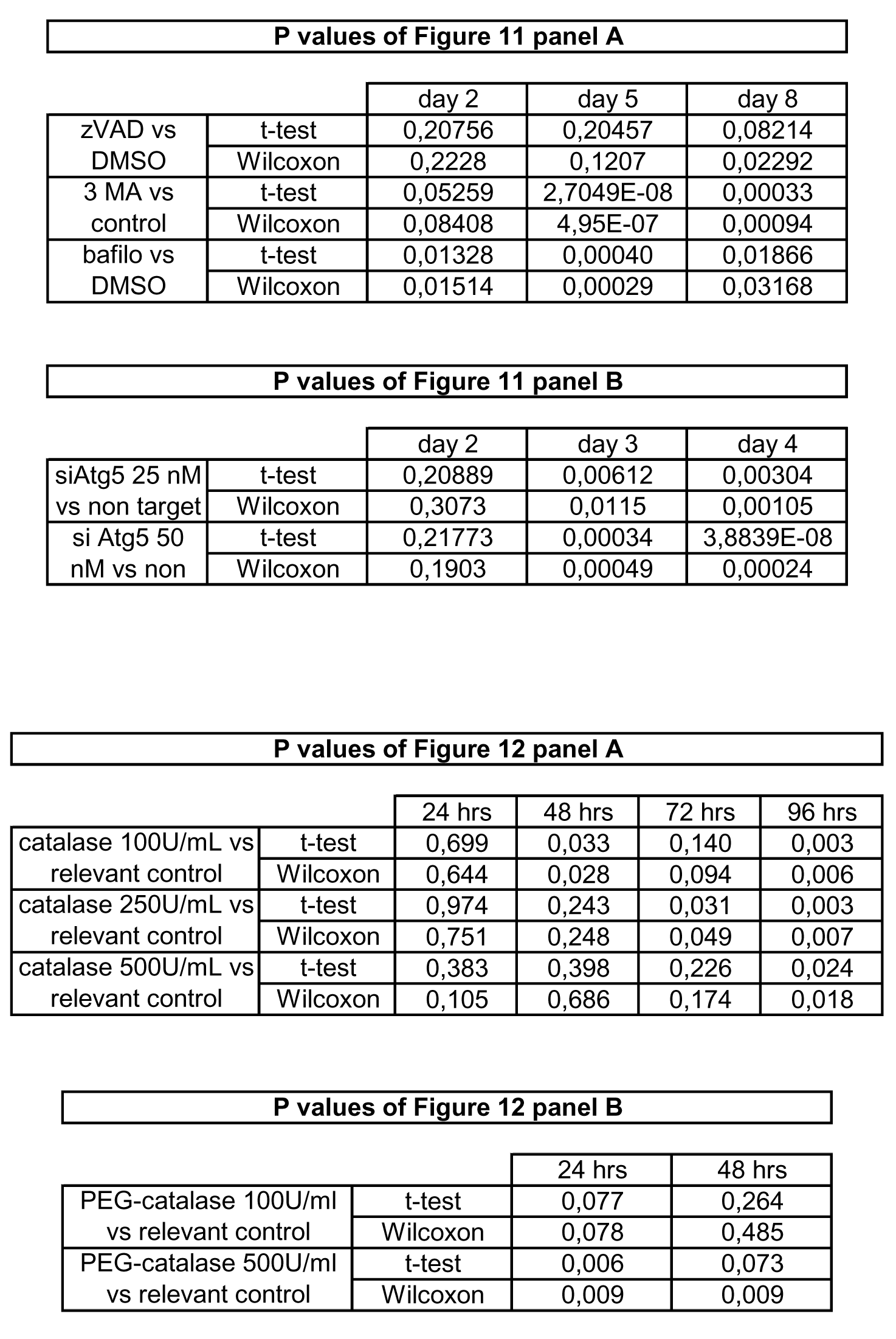

Supplement: Figure S6 — Statistical analysis of the results of Figures 11 and 12. (0.61 MB TIF) [file pone.0012712.s006.tif]
